# A multicentre, open-label, phase-I/randomised phase-II study to evaluate safety, pharmacokinetics, and efficacy of nintedanib vs. sorafenib in European patients with advanced hepatocellular carcinoma

**DOI:** 10.1038/s41416-018-0051-8

**Published:** 2018-03-22

**Authors:** D. H. Palmer, Y. T. Ma, M. Peck-Radosavljevic, P. Ross, J. Graham, L. Fartoux, A. Deptala, M. Studeny, D. Schnell, J. Hocke, A-B. Loembé, T. Meyer

**Affiliations:** 1 Department of Molecular and Clinical Cancer Medicine, University of Liverpool, Liverpool, United Kingdom; 2 Clatterbridge Cancer Centre, Bebington, United Kingdom; 3 School of Cancer Sciences, University of Birmingham, Birmingham, United Kingdom; 4 Department of Gastroenterology & Hepatology, Medizinische Universität Wien, Vienna, Austria; 5 King’s College Hospital NHS Foundation Trust, London, United Kingdom; 6 Beatson West of Scotland Cancer Centre and University of Glasgow, Glasgow, United Kingdom; 7 Hôpital Saint-Antoine, Paris, France; 8 Central Clinical Hospital of the Ministry of Interior, Department of Oncology and Hematology, Medical University of Warsaw, Warsaw, Poland; 9 Boehringer Ingelheim, Pharma GmbH & Co. KG, Biberach, Germany; 10 Boehringer Ingelheim B.V., Alkmaar, The Netherlands; 11 UCL Cancer Institute, University College London, London, United Kingdom

**Keywords:** Phase II trials, Hepatocellular carcinoma

## Abstract

**Background:**

This multicentre, open-label, phase-I/randomised phase-II trial evaluated safety, pharmacokinetics, maximum-tolerated-dose (MTD) per dose-limiting toxicities (DLTs), and efficacy of nintedanib vs. sorafenib in European patients with unresectable advanced hepatocellular carcinoma (aHCC).

**Methods:**

Phase I: Patients were stratified into two groups per baseline aminotransferase/alanine aminotransferase and Child-Pugh score; MTD was determined. Phase II: Patients were randomised 2:1 to nintedanib (MTD) or sorafenib (400-mg bid) in 28-day cycles until intolerance or disease progression. Time-to-progression (TTP, primary endpoint), overall survival (OS) and progression-free survival (PFS) were determined.

**Results:**

Phase-I: no DLTs observed; nintedanib MTD in both groups was 200 mg bid. Phase-II: patients (*N *= 93) were randomised to nintedanib (*n *= 62) or sorafenib (*n* = 31); TTP was 5.5 vs. 4.6 months (HR = 1.44 [95% CI, 0.81–2.57]), OS was 11.9 vs. 11.4 months (HR = 0.88 [95% CI, 0.52–1.47]), PFS was 5.3 vs. 3.9 months (HR = 1.35 [95% CI, 0.78–2.34]), respectively (all medians). Dose intensity and tolerability favoured nintedanib. Fewer patients on nintedanib (87.1%) vs. sorafenib (96.8%) had drug-related adverse events (AEs) or grade ≥ 3 AEs (67.7% vs. 90.3%), but more patients on nintedanib (28 [45.2%]) had AEs leading to drug discontinuation than did those on sorafenib (7 [22.6%]).

**Conclusions:**

Nintedanib may have similar efficacy to sorafenib in aHCC.

## Introduction

Hepatocellular carcinoma (HCC) is a hyper-vascular tumour^1^ involving dysregulation of several angiogenic growth factors such as vascular endothelial growth factor (VEGF),^2–4^ epidermal growth factor (EGF),^2–4^ platelet-derived growth factor (PDGF)^2–4^ and fibroblast growth factor (FGF) .^5^ Sorafenib is a small-molecule kinase inhibitor of VEGFR, PDGFR, RAF, c-KIT and FLT-3 ^6^, and is the first approved targeted drug worldwide for first-line treatment in advanced HCC. Phase III trials in Western ^7^ and Asian patients ^6^ have shown it to significantly improve overall survival (OS) compared with placebo. However, the effect of sorafenib in HCC is modest, with median survival prolonged by 2–3 months compared with placebo.^6,7^

Primary resistance to VEGF-targeted therapy, such as sorafenib, may occur through a variety of mechanisms,^8,9^ which may be overcome by targeting multiple pathways either through combination regimens or drugs with multiple targets .^2^ Nintedanib is an oral, small-molecule, triple angiokinase inhibitor of VEGFR1-3, PDGFRα and β, FGFR1-3, Flt-3, Lck, Lyn and Src,^10^ with anti-tumour and anti-angiogenic activity in preclinical models of HCC .^11^ Nintedanib is metabolised in the liver ^12^, and it is not clear if liver impairment influences nintedanib’s metabolism, elimination, tolerability and maximum-tolerated dose (MTD). Because of this, the published guidelines for HCC drug development recommend initial phase-I trials to determine the MTD of a novel agent in the context of chronic liver disease followed by randomised phase-II trials with time to progression (TTP) as the primary endpoint .^13^ Nintedanib for HCC has been developed in accordance with these guidelines. We report the results of a multicentre, open-label, randomised phase-I/II trial that evaluated the MTD, pharmacokinetics (PK), efficacy and safety of nintedanib vs. sorafenib in European patients with advanced HCC, who did not have prior systemic treatment (NCT01004003; 1199.37). Since different risk factors between Western and Asian populations, such as incidences of hepatitis B virus (HBV) and hepatitis C virus (HCV), may influence responses to targeted therapies, an identically designed study was conducted in parallel in Asian patients ^14,15^ and is reported separately (NCT00987935; 1199.39).

## Materials and methods

### Study population and eligibility

Key inclusion criteria included patients aged ≥18 years with advanced HCC not amenable to curative/locoregional therapy and with ≥1 measurable lesion by Response Evaluation Criteria in Solid Tumours (RECIST) v1.0 (for phase-II); Eastern Cooperative Oncology Group (ECOG) performance score (PS) ≤2; Child–Pugh score 5–6 (for phase-II) and alanine/aspartate aminotransferase (ALT/AST) levels ≤2 times upper limit of normal (ULN) (for phase-II and phase-I group I; phase-I group II had ALT or AST >2–≤5 times ULN or Child–Pugh score 7); >4 weeks since most recent local therapy; no prior systemic therapy for HCC; no history of other malignancy within the past 3 years; and life expectancy ≥12 weeks. Additional inclusion criteria are listed in the protocol. The trial protocol was reviewed by the institutional review board of each participating centre and conforms to the Helsinki Declaration. All patients provided written informed consent (ClinicalTrials.gov registration: NCT01004003).

### Study design and treatment

The primary endpoint for phase-I was nintedanib MTD determination in terms of dose-limiting toxicities (DLTs) occurring during the MTD determination period (i.e. within the first 28 days of therapy). Pharmacokinetics of nintedanib and its main metabolites BIBF 1202 and BIBF 1202 glucuronide were assessed as an exploratory endpoint. In view of the observed DLT of transaminitis in the non-HCC oncology population, patients were stratified into two groups according to their ALT/AST and Child–Pugh score at baseline. Group I contained patients with ALT and AST ≤2 times ULN and Child–Pugh score 5–6, while group II contained patients with ALT or AST >2–≤5 times ULN or Child–Pugh score 7. An expansion cohort of group II patients was started. MTD determination methods and DLT definitions for nintedanib appear in supplementary Data.

Extensive plasma and urine sampling was performed during the first treatment cycle to describe the PK characteristics of nintedanib and its main metabolites BIBF 1202 and BIBF 1202 glucuronide. Assessments were performed according to the schedule shown in the protocol. Antiviral treatment was recommended for patients with chronic HBV infection.

For phase-II, 93 patients were randomised in a 2:1 ratio to receive nintedanib 200 mg bid (*n* = 62) or sorafenib 400 mg bid (*n* = 31) continuously, in 28-day cycles, until intolerable AEs or disease progression (PD). A randomised design was used in favour of performing an uncontrolled trial, which was common at the time the trial was conceived, to permit a contemporaneous comparison against sorafenib. A 2:1 randomisation ratio was chosen to generate more data on nintedanib, especially regarding safety, because data for sorafenib were already extensively defined. Treatment beyond PD was allowed at the discretion of the investigator if there was perceived ongoing clinical benefit.

Randomisation was performed by an integrated response system using a validated randomisation number-generating system. Randomisation was stratified by presence of extrahepatic spread (EHS) and macrovascular invasion (MVI) (EHS and/or MVI present *vs*. both absent). Full details of randomisation, as well as the dose reduction scheme and trial procedures and assessments, are shown in the protocol and supplementary Data.

### Study outcomes

The primary endpoints for phase-I and II, respectively, were determination of the MTD in terms of DLTs and TTP by central independent review (CIR) according to RECIST v1.0 (which were the recommended criteria when the protocol was written). Main secondary endpoints for phase-II were objective tumour response (OR) according to RECIST v1.0 assessed by CIR, defined as a best response of complete response (CR) or partial response (PR); progression-free survival (PFS) assessed by CIR; and OS. Definitions and further outcome measures are described in supplementary Data.

The primary analysis for efficacy and safety was performed after 80% of all planned patients (77% of all included patients) had an investigator-assessed TTP event.

Safety and tolerability were assessed based on the incidence and severity of adverse events (AEs) according to CTCAE v3.0, laboratory abnormalities, physical examination, ECOG PS, vital signs and electrocardiogram.

### Pharmacokinetic sampling

See supplementary Data for details.

### Statistical analysis

All analyses were exploratory; any statistical tests were performed only to provide a statistical framework from which to view the results and aid planning of further studies. The sample size was selected to provide a high probability of recording a numerically positive treatment effect of nintedanib vs. sorafenib. By assuming median TTPs of 9 months for nintedanib and 6 months for sorafenib (hazard ratio [HR] 0.67) to represent a clinically significant benefit compared with sorafenib, the probability of observing any numerically positive treatment effect on TTP (i.e. an estimated HR for TTP between nintedanib and sorafenib of <1) is around 93% for 90 patients (2:1 randomisation, 8 months accrual and 14 months follow-up). See supplementary Data for further details.

WinNonlin 5.2 (Certara, Princeton, NJ, USA) and SAS Version 9.2 (SAS, Cary, NC, USA) were used for all statistical and PK analyses.

## Results

### Patient characteristics

Between November 11, 2009 and September 24, 2013, a total of 32 patients (13 in group I and 19 in group II) were recruited in phase-I from 4 centres in 2 European countries. Six patients were screened, but did not enter the trial. Supplementary Table S1 summarises patient demographics and baseline disease characteristics; supplementary Table S2 shows the patient disposition.

For phase-II, of the 132 enroled patients, 93 were randomly assigned from September 19, 2011 until November 14, 2012, with all receiving at least one dose of nintedanib (*n* = 62) or sorafenib (*n* = 31; supplementary Figure S1). There were 28 investigational sites in eight European countries involved in enrolment, of which 26 randomised and treated patients. With the exception of no evidence of parenchymal liver disease (24.2% in the nintedanib group vs. 3.2% in the sorafenib group), and HBV-related liver disease (6.5% in the nintedanib group vs. 22.6% in the sorafenib group), baseline characteristics were well-balanced between the two treatment groups. Approximately three quarters of all patients in both groups were Barcelona Clinic Liver Cancer (BCLC) stage C (and the remainder mostly stage B). Table 1 summarises baseline patient characteristics.

**Table 1:** Phase-II patient baseline demographics and clinical characteristics^a^

Characteristic	Nintedanib, 200 mg bid (*n* = 62)	Sorafenib, 400 mg bid (*n* = 31)	Total (*N* = 93)
Median age, years (range)	66 (34–86)	64 (28–83)	66 (28–86)
Male sex, *n* (%)	48 (77.4)	26 (83.9)	74 (79.6)
Race, *n* (%)			
Indian	0 (0.0)	3 (9.7)	3 (3.2)
Taiwanese or Chinese	0 (0.0)	1 (3.2)	1 (1.1)
Black	0 (0.0)	1 (3.2)	1 (1.1)
Caucasian	57 (91.9)	24 (77.4)	81 (87.1)
Missing	5 (8.1)	2 (6.5)	7 (7.5)
Median time since diagnosis, months (range)	2.53 (0–101.4)	2.76 (0.2–77.5)	2.53 (0–101.4)
ECOG PS, *n* (%)			
0	32 (51.6)	18 (58.1)	50 (53.8)
1	28 (45.2)	10 (32.3)	38 (40.9)
2	2 (3.2)	3 (9.7)	5 (5.4)
Child–Pugh score, *n* (%)			
5	42 (67.7)	23 (74.2)	65 (69.9)
6	19 (30.6)	8 (25.8)	27 (29.0)
7^b^	1 (1.6)	0 (0.0)	1 (1.1)
BCLC stage, *n* (%)			
0	0 (0.0)	1 (3.2)	1 (1.1)
A	1 (1.6)	0 (0.0)	1 (1.1)
B	15 (24.2)	7 (22.6)	22 (23.7)
C	45 (72.6)	23 (74.2)	68 (73.1)
D	1 (1.6)	0 (0.0)	1 (1.1)
MVI, *n* (%)	22 (35.5)	9 (29.0)	31 (33.3)
EHS, *n* (%)	40 (64.5)	21 (67.7)	61 (65.6)
Location of EHS, *n* (%)			
Bone	6 (9.7)	5 (16.1)	11 (11.8)
Lung	16 (25.8)	6 (19.4)	22 (23.7)
Lymph	26 (41.9)	9 (29.0)	35 (37.6)
Other	11 (17.7)	7 (22.6)	18 (19.4)
Aetiology of parenchymal liver disease, n (%)			
Alcohol related	10 (16.1)	3 (9.7)	13 (14.0)
HBV related	4 (6.5)	7 (22.6)	11 (11.8)
HCV related	13 (21.0)	8 (25.8)	21 (22.6)
HBV + HCV related	0 (0.0)	0 (0.0)	0 (0.0)
Unknown	23 (37.1)	8 (25.8)	31 (33.3)
Other	12 (19.4)	5 (16.1)	17 (18.3)
Parenchymal liver disease, n (%)			
Chronic hepatitis	8 (12.9)	5 (16.1)	13 (14.0)
Steatofibrosis	3 (4.8)	2 (6.5)	5 (5.4)
Cirrhosis	29 (46.8)	20 (64.5)	49 (52.7)
No evidence	15 (24.2)	1 (3.2)	16 (17.2)
Unknown	6 (9.7)	3 (9.7)	9 (9.7)
Other	1 (1.6)	0 (0.0)	1 (1.1)
Type of local therapy, *n* (%)			
Complete surgical resection	9 (14.5)	3 (9.7)	12 (12.9)
RFA	2 (3.2)	0 (0.0)	2 (2.2)
PEI	0 (0.0)	0 (0.0)	0 (0.0)
TACE	19 (30.6)	10 (32.3)	29 (31.2)
RT	1 (1.6)	0 (0.0)	1 (1.1)
Other	2 (3.2)	4 (12.9)	6 (6.5)
Stratification group, n (%)			
I: EHS and/or MVI present	49 (79.0)	23 (74.2)	72 (77.4)
II: EHS and MVI both absent	13 (21.0)	8 (25.8)	21 (22.6)

*BCLC* Barcelona Clinic Liver Cancer, *ECOG PS* Eastern Cooperative Oncology Group performance status, *EHS* extrahepatic spread, *HBV* hepatitis B, *HCV* hepatitis C, *MVI* macrovascular invasion, *PEI* percutaneous ethanol injection, *RFA* radiofrequency ablation, *RT* radiotherapy, *TACE* transarterial chemoembolisation.

^a^α-fetoprotein (AFP) groups are not shown because there were too many missing values because a lot of investigative sites measured activated AFP instead of AFP and there is no transformation of the values available.

^b^This patient with a Child–Pugh score of 7 in the nintedanib group was a protocol deviation

### Patient disposition and treatment exposure

The median (range) duration of treatment in phase-I was 11.3 (0.07–40.5) months in group I vs. 2.14 (0.03–14.1) months in group II. Median dose intensity for all dose cohorts was 100%, with the exception of the 200 mg cohort in group I, which was 99.3%. Nintedanib dose reductions and exposure for phase-I are summarised in supplementary Table S3.

In phase-II, the median durations of nintedanib and sorafenib treatments were similar (5.40 months and 5.42 months, respectively). However, the mean treatment duration was nominally longer in the nintedanib (8.08 months) vs. the sorafenib (7.28 months) group. Mean dose intensity was also higher for nintedanib (96.4% in the nintedanib vs. 84.8% in the sorafenib group).

### Determination of MTD

There were no DLTs during the MTD determination period in either group at any dose up to the prespecified maximum of 200 mg bid. Thus, the recommended phase-II dose was determined to be 200 mg bid for group I and II patients. DLTs experienced after the MTD determination period are described in supplementary Data.

### Pharmacokinetics

PK results appear in supplementary Data. In brief, nintedanib was rapidly absorbed followed by at least biphasic disposition kinetics (supplementary Figure S2). Maximum plasma concentrations were achieved around a median of 2 h and the gMean half-life ranged between 18.3 and 33.0 h (supplementary Table S4). We observed a trend towards an increased gMean exposure to nintedanib, BIBF 1202 and BIBF 1202 glucuronide in group II patients compared with those of group I (supplementary Table S5). However, the range of individual values strongly overlapped when data from both groups were compared.

### Phase-II efficacy

#### Primary endpoint

**Fig. 1:**
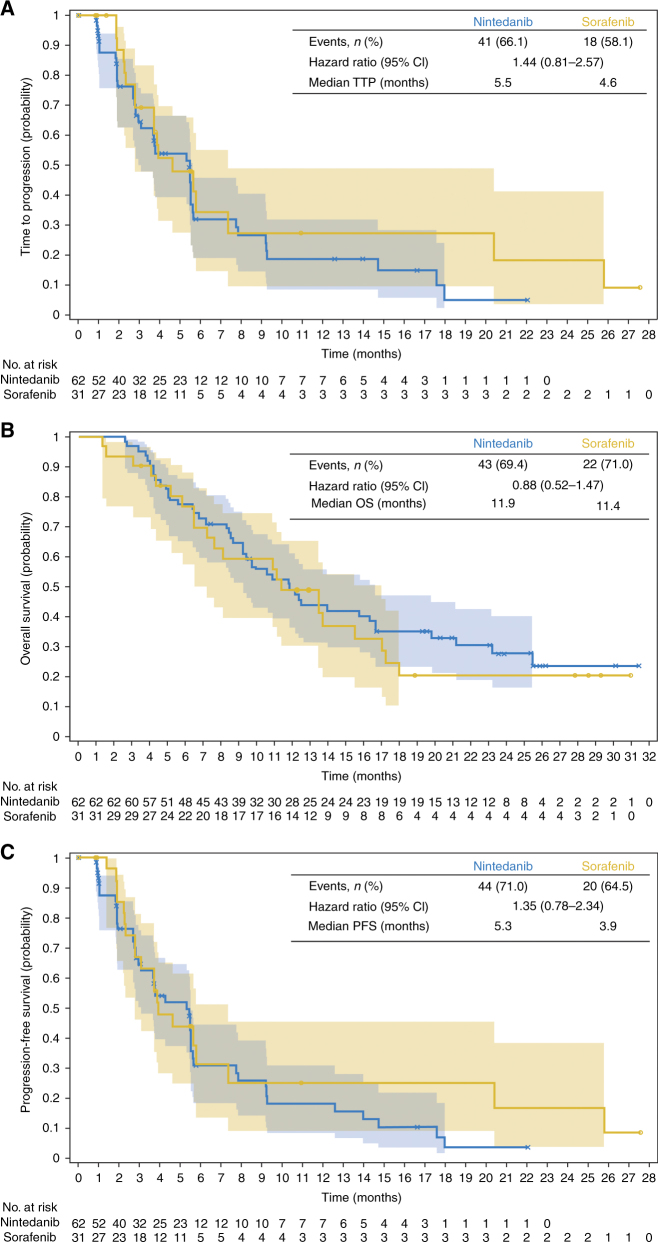
Phase-II Kaplan-Meier curves of (**A**) time to tumour progression by central independent review assessed according to Response Evaluation Criteria for Solid Tumours (RECIST) v1.0, (**B**) overall survival, and (**C**) progression-free survival by central independent review assessed according to RECIST v1.0. OS, overall survival; PFS, progression-free survival; TTP, time to progression

The median TTP according to RECIST v1.0 by CIR was 5.5 months (95% CI, 3.0–5.6) in the nintedanib group vs. 4.6 months (95% CI, 2.8–7.4) in the sorafenib group; HR = 1.44 (95% CI, 0.81–2.57) (Fig. 1a).

#### Secondary endpoints

Median OS was 11.9 months (95% CI, 9.2–16.7) in the nintedanib group vs. 11.4 months (95% CI, 6.5–17.0) in the sorafenib group; HR = 0.88 (95% CI, 0.52–1.47; Fig. 1b).

Median PFS according to RECIST v1.0 by CIR was 5.3 months for nintedanib (95% CI, 3.0–5.5) vs. 3.9 months for sorafenib (95% CI, 2.8–7.4); HR = 1.35 (95% CI, 0.78–2.34; Fig. 1c). Radiological response according to RECIST and mRECIST by CIR is summarised in supplementary Table S6, including the secondary endpoint OR according to RECIST by CIR. The best percentage change from baseline in target lesions by CIR for nintedanib and sorafenib are shown in supplementary Figure S3A and Figure S3B, respectively. One patient on nintedanib had a PR of 16.0 months duration according to RECIST by CIR; two patients on sorafenib had a PR that lasted for 1.9 months and 2.9 months. According to mRECIST by CIR, there were seven patients on nintedanib and six patients on sorafenib with a PR. At the cut-off date of July 15, 2014, two patients on nintedanib and one on sorafenib continued treatment, with one patient on nintedanib continuing despite progression by RECIST. This patient continued on treatment beyond progression for 169 days.

One patient underwent resection following treatment on nintedanib and remains in complete remission; this was a non-cirrhotic man who presented with a poorly vascularised HCC that did not respond to transarterial chemoembolisation. He was then offered systemic therapy and consented to participate in this study. Following a long period of disease control and multidisciplinary team review, he underwent surgical resection.

#### Exploratory endpoints

Median TTP as assessed by mRECIST by CIR in the nintedanib group was 5.5 months (95% CI, 3.1–5.6) vs. 5.5 months in the sorafenib group (95% CI, 2.8–7.4); HR = 1.59 (95% CI, 0.88–2.85) and median TTP by investigator assessment in the nintedanib group was 5.5 months (95% CI, 3.1–7.4) vs. 3.8 months in the sorafenib group (95% CI, 3.5 to 5.7); HR = 1.05 (95% CI, 0.63–1.76).

Median time to treatment failure was 3.7 months for nintedanib (95% CI, 2.7–5.5) vs. 3.7 months for sorafenib (95% CI, 2.3–5.7); HR = 1.27 (95% CI, 0.79–2.02). Subgroup analysis according to performance status, aetiology of parenchymal liver disease, baseline hypertension and EHS or MVI suggest no differences in TTP between the treatment arms, but subgroups were small (data not shown).

### Safety and tolerability

AEs occurring during the phase-I portion are reported in supplementary Data. For the phase-II portion, fewer patients on nintedanib (87.1%) vs. sorafenib (96.8%) had investigator-defined drug-related AEs and fewer patients on nintedanib (67.7%) experienced grade 3 or higher AEs compared with those on sorafenib (90.3%). AEs occurring during the phase-II portion are summarised in supplementary Table S7. A total of 25 patients had AEs leading to dose reduction; 12 (19.4%) in the nintedanib and 13 (41.9%) in the sorafenib group (supplementary Table S8). AEs leading to drug discontinuation occurred in 28 (45.2%) patients in the nintedanib group and 7 (22.6%) patients in the sorafenib group (supplementary Table S8). None of the patients taking sorafenib had grade 4 or 5 AEs that led to drug discontinuation, whereas for nintedanib four patients each had grade 4 AEs of hypertensive crisis, oesophageal varices haemorrhage, fatigue, asthenia and decreased performance status, plus one patient each with grade 5 general physical health deterioration and PD that led to drug discontinuation (none of which were drug related except for hypertensive crisis, fatigue and asthenia).

AEs associated with VEGF pathway inhibition, and other any-grade AEs of special interest, included (for nintedanib vs. sorafenib) specific liver-related investigations (24.2% vs. 25.8%), bleeding (29.0% vs. 22.6%), rash (21.0% vs. 38.7%), hypertension (14.5% vs. 9.7%), cutaneous adverse reactions (4.8% vs. 19.4%), thromboembolic events (1.6% vs. 12.9%) and gastrointestinal (GI) perforation (0% vs. 3.2%). No arterial thromboembolic events were reported. Supplementary Table S9 shows grade ≥3 AEs of special interest occurring in all the dose cohorts for groups I and II.

Frequently occurring any-grade AEs are summarised in Table 2. Nausea (48.4% vs. 29.0%), vomiting (38.7% vs. 29.0%) and upper abdominal pain (25.8% vs. 12.9%) occurred >10% more frequently with nintedanib compared with sorafenib. Palmar–plantar erythrodysesthesia syndrome (35.5% vs. 1.6%), alopecia (35.5% vs. 4.8%) and rash (22.6% vs. 9.7%) were more frequent with sorafenib compared with nintedanib (Table 2). Grade ≥3 palmar–plantar erythrodysesthesia syndrome occurred in 22.6% of patients on sorafenib and no patients on nintedanib. AEs of ≥grade 3 occurring in ≥5% of patients are summarised in Table 2. There were 9 (14.5%) patients on nintedanib and 3 (9.7%) on sorafenib for whom AEs led to death; this was related to tumour progression for both drugs for all but one patient in the nintedanib group who died of interstitial lung disease, for which a causal relationship to the study drug could not be excluded.

**Table 2:** Most frequently reported (patient level) adverse events by primary system organ class and preferred term (occurring at any grade in ≥20% or at grade ≥3 in ≥5% patients in either treatment group) during the phase-II portion

Adverse event (SOC and PT)	Nintedanib, 200 mg bid (*n* = 62)		Sorafenib, 400 mg bid (*n* = 31)	
	All grades, *n* (%)	Grade ≥3, *n* (%)	All grades, *n* (%)	Grade ≥3, *n* (%)
Patients with any adverse event	62 (100)	42 (67.7)	31 (100)	28 (90.3)
Blood and lymphatic system disorders	10 (16.1)	5 (8.1)	8 (25.8)	6 (19.4)
Anaemia	6 (9.7)	4 (6.5)	1 (3.2)	1 (3.2)
Thrombocytopenia	2 (3.2)	1 (1.6)	4 (12.9)	3 (9.7)
Gastrointestinal disorders	60 (96.8)	16 (25.8)	29 (93.5)	3 (9.7)
Diarrhoea	44 (71.0)	8 (12.9)	21 (67.7)	1 (3.2)
Nausea	30 (48.4)	1 (1.6)	9 (29.0)	0 (0.0)
Vomiting	24 (38.7)	2 (3.2)	9 (29.0)	0 (0.0)
Abdominal pain	16 (25.8)	0 (0.0)	9 (29.0)	1 (3.2)
Abdominal pain upper	16 (25.8)	0 (0.0)	4 (12.9)	0 (0.0)
General disorders and administration site conditions	44 (71.0)	11 (17.7)	21 (67.7)	4 (12.9)
Fatigue	35 (56.5)	7 (11.3)	10 (32.3)	1 (3.2)
Investigations	24 (38.7)	14 (22.6)	17 (54.8)	11 (35.5)
AST increased	11 (17.7)	7 (11.3)	5 (16.1)	1 (3.2)
ALT increased	8 (12.9)	5 (8.1)	3 (9.7)	2 (6.5)
Metabolism and nutrition disorders	26 (41.9)	4 (6.5)	18 (58.1)	4 (12.9)
Decreased appetite	23 (37.1)	1 (1.6)	13 (41.9)	1 (3.2)
Neoplasms benign, malignant and unspecified (including cysts and polyps)	6 (9.7)	4 (6.5)	4 (12.9)	3 (9.7)
Malignant neoplasm progression	2 (3.2)	2 (3.2)	3 (9.7)	3 (9.7)
Nervous system disorders	20 (32.3)	7 (11.3)	16 (51.6)	2 (6.5)
Hepatic encephalopathy	5 (8.1)	5 (8.1)	2 (6.5)	1 (3.2)
Lethargy	5 (8.1)	0 (0.0)	8 (25.8)	1 (3.2)
Skin and subcutaneous tissue disorders	27 (43.5)	2 (3.2)	25 (80.6)	11 (35.5)
Rash	6 (9.7)	0 (0.0)	7 (22.6)	1 (3.2)
Alopecia	3 (4.8)	0 (0.0)	11 (35.5)	0 (0.0)
Palmar–plantar erythrodysesthesia syndrome	1 (1.6)	0 (0.0)	11 (35.5)	7 (22.6)
Skin reaction	1 (1.6)	1 (1.6)	3 (9.7)	2 (6.5

*ALT* alanine aminotransferase, *AST* aspartate aminotransferase, *PT* preferred term, *SOC* system organ class

## Discussion

This trial showed that nintedanib had antitumour activity and suggests it had similar efficacy with sorafenib, with a tolerable and different AE profile, in mainly Caucasian patients with advanced HCC. The trial design complied with European Association for the Study of the Liver (EASL) recommendations for HCC trials ^16^, as well as those for clinical oncology trials,^17^ using a randomised design with an active comparator, and was conducted to determine whether a high probability of success might be indicated for phase III trials, rather than to obtain definitive efficacy information. There were no pre-planned criteria for progression to a phase III trial, however. The trial used outcome measures recommended for use in phase-II trials in HCC,^13^ and as is common with parallel-group designs was open-label to better monitor safety on an ongoing basis and because of the different safety profiles and different rules for dose interruptions and reductions between the nintedanib and sorafenib groups. A comparable trial with nintedanib in Asian patients found similar results.^14,15^

In the phase-I portion, the area under the curve for nintedanib was broadly comparable to that found in other malignancies.^18,19^ Nintedanib is metabolised and mainly excreted via the liver ^12^ such that liver impairment may influence nintedanib pharmacokinetics. Thus, an exploratory objective of this trial was to describe the effect of liver function assessed by AST/ALT plasma concentrations at study baseline (by allocation of patients to group I or II) and Child–Pugh classification on the PK parameters of nintedanib and its metabolites. Patients were stratified into two groups according to baseline liver function and the MTD was determined to be the same (200 mg bid) for both groups with no significant differences in PK parameters. No DLTs occurred during the MTD determination period. The main DLTs occurring after the MTD determination period were reversible liver enzyme elevations. In the phase-II portion of the trial, a higher proportion of patients in the sorafenib group had severe AEs, drug-related AEs and AEs leading to dose reductions compared with patients in the nintedanib group, while a higher proportion of patients in the nintedanib group (45.2%) had AEs leading to drug discontinuation compared with those in the sorafenib group (22.6%). Nonetheless, despite this high rate of nintedanib discontinuation, the duration of nintedanib treatment was longer than that of sorafenib and the dose intensity of nintedanib was better, indicating that nintedanib is reasonably tolerated relative to sorafenib. Approximately a quarter of patients on nintedanib experienced GI AEs of grade 3 or higher. This rate was higher than that with sorafenib (9.7%), underscoring the importance of close clinical monitoring for GI events, especially given the large impact of GI toxicities on quality of life. The higher rates of variceal bleeding with nintedanib, including that leading to drug discontinuation, suggest baseline endoscopy and control of varices could be a prudent strategy prior to starting nintedanib. Any-grade drug-related AEs for sorafenib were generally similar to those in the SHARP trial,^7^ although occurring at higher rates in the present trial, particularly for diarrhoea (67.7% vs. 39% in SHARP) and palmar–plantar erythrodysesthesia syndrome (35.5% vs. 21% in SHARP).

Allowing for small patient numbers and wide confidence intervals, the efficacy in terms of TTP and OS for sorafenib in this trial is broadly comparable to that reported in the SHARP trial, which was also conducted in a predominantly European population.

Limitations of this trial include the fact that correlations between TTP and PFS with OS still need to be established (a problem common to all trials in the setting of HCC). Because the trial was not blinded or powered to detect a significant difference in OS, further blinded, randomised studies would be required to interrogate potential survival benefits of nintedanib over sorafenib. The patients included in the phase-II portion were untreated, first-line patients. Given the limited benefit of sorafenib in this setting, documented progressive disease before inclusion could have been a better way to identify potential differences between sorafenib and nintedanib. Data on subsequent post-progression treatment were not mandated, such that its impact on OS could not be assessed. In contrast with sorafenib, nintedanib has activity inhibiting FGF receptor signalling. Although the FGF receptor status of patients was not assessed in this study, future studies could examine the FGF status of responders to nintedanib.

In conclusion, the current trial suggests nintedanib may have similar efficacy to sorafenib in patients with advanced HCC, with a tolerable and different safety profile, but with higher VEGF-related toxicity. The results suggest that nintedanib could be a suitable partner for combination studies in HCC.

## Electronic supplementary material

Supplementary Data(DOCX 35 kb)

Supplementary Figure S1(TIF 1099 kb)

Supplementary Figure S2(TIF 745 kb)

Supplementary Figure S3(TIF 405 kb)

Supplementary Table S1(DOCX 33 kb)

Supplementary Table S2(DOCX 25 kb)

Supplementary Table S3(DOCX 25 kb)

Supplementary Table S4(DOCX 29 kb)

Supplementary Table S5(DOCX 27 kb)

Supplementary Table S6(DOCX 26 kb)

Supplementary Table S7(DOCX 26 kb)

Supplementary Table S8(DOCX 47 kb)

Supplementary Table S9(DOCX 32 kb)
