# Supplementary material for: A multicentre, open-label, phase-I/randomised phase-II study to evaluate safety, pharmacokinetics, and efficacy of nintedanib vs. sorafenib in European patients with advanced hepatocellular carcinoma
Source: Br J Cancer. 2018 Mar 22;118(9):1162–8. doi: 10.1038/s41416-018-0051-8 (PMC5943284; doi:10.1038/s41416-018-0051-8)
Supplement: Supplementary file 1 — Supplementary Data(DOCX 35 kb) [file 41416_2018_51_MOESM1_ESM.docx]

**Supplementary Data**

**Patients and methods**

***Study Design and Treatment***

Dose escalation followed a standard 3 + 3 design, starting at 50 mg bid (group II) or 100 mg bid (group I) and escalating up to 200 mg bid in 50-mg bid intervals. These starting doses represent 20% and 40%, respectively, of the MTD (250 mg bid) determined in patients with normal liver function to provide adequate safety margins for HCC patients who may have reduced capability to metabolize and excrete nintedanib. Patients were administered nintedanib orally for treatment courses of 28 days and were assessed for retreatment eligibility on the day 29 visit. If a patient experienced a DLT, treatment with nintedanib was interrupted and could be resumed at a reduced dose level after re-starting criteria were met. Patients with clinical benefit and no undue toxicity continued treatment in repeated treatment courses. The MTD for groups I and II were determined separately.

The MTD was defined as the highest dose of nintedanib at which 0/3 or <2/6 patients experienced a DLT. DLTs during the first treatment course were used for MTD determination. However, all unusual or unexpected toxicities occurring throughout the treatment were also considered for the purpose of confirming the MTD and recommended phase II dose. Dose escalation stopped at the highest prespecified dose level if the MTD was not otherwise defined by a DLT. DLTs for nintedanib were defined as nintedanib-related Common Terminology Criteria for Adverse Events (CTCAE) version 3.0 grade 4 thrombocytopenia of any duration; nintedanib-related CTCAE grade 4 neutropenia lasting for 8 or more days; nintedanib-related febrile neutropenia of any duration; nintedanib-related CTCAE grade 3 or 4 non-hematologic toxicity (except alopecia, untreated vomiting, or diarrhea, and liver toxicity); or nintedanib-related liver toxicity except isolated gamma glutamyltransferase (γ-GT) elevation with no corresponding increase in ALT/AST/alkaline phosphatase (ALP). For patients with normal liver enzymes at baseline the DLT definition (corresponding to grade 3 toxicity according to CTCAE) for AST/ALT/ALP was elevation >5×ULN and for total bilirubin was elevation >3×ULN. For patients with elevated liver enzymes at baseline, the DLT definition for AST/ALT/ALP was elevation >(baseline value + 4×ULN) and for total bilirubin was elevation >3×ULN.

Randomization was performed by an integrated response system using a validated randomization number–generating system implemented by Almac (Craigavon, UK). The random code was generated by the Boehringer Ingelheim randomization group and checked by a trial-independent statistician. For patients in the nintedanib group who had predefined AEs, two dose reductions were allowed according to a prespecified scheme (from 200 mg bid to 150 mg bid and thereafter to 100 mg bid) with no subsequent dose escalation upon resolution of the AE; for patients in the sorafenib group, doses were delayed or reduced for clinically significant hematologic and other AEs according to a prespecified scheme as shown in the Appendix. Tumor assessment by RECIST v1.0 was performed every 4 weeks for the first 16 weeks after the start of treatment and every 8 weeks thereafter. The baseline scan was performed within 3 weeks prior to treatment. Treatment decisions were based on investigator assessment. Assessment by central independent review (CIR) was pre-planned and prospective per patient but did not influence treatment decisions. Full details of trial procedures and assessments appear in the protocol (online only). Patients who interrupted treatment for 14 or more consecutive days except for interruption due to AEs were considered non-compliant.

***Study Outcomes***

The primary endpoint was TTP by CIR, defined as the time from randomization to disease progression according to RECIST v1.0. Main secondary endpoints were objective tumor response (OR) according to RECIST v1.0 assessed by CIR, defined as a best response of complete response (CR) or partial response (PR); progression-free survival (PFS) assessed by CIR, defined as time from randomization to disease progression according to RECIST v1.0 or death, whichever occurred earlier; and OS, defined as time from randomization until death. Additional analyses included time to treatment failure, defined as time from randomization to earliest date of disease progression by RECIST v1.0 by CIR, death, or date of stopping study medication because of AEs; TTP by investigator assessment according to RECIST v1.0; OR and TTP according to modified RECIST (mRECIST) for HCC criteria [[16](#_ENREF_16)] (CIR only); and safety of nintedanib as indicated by incidence and intensity of AEs according to CTCAE v3.0 and laboratory evaluations.

***Pharmacokinetic Sampling***

For quantification of nintedanib, BIBF 1202, and BIBF 1202 glucuronide plasma concentrations, 5 mL of blood was taken from a forearm vein in an EDTA-anticoagulant blood drawing tube at the following time points in the first treatment course (day 15 to 16): Immediately prior to swallowing the dose of nintedanib (predose) and 1, 2, 3, 4, 5, 7, 10, 12 (allowed time frame 11 to 13 hours), and 24 hours (allowed time frame 22 to 26 hours) after drug administration on day 15 (the evening dose on day 15 was omitted).

Urine was sampled in the following intervals: 0 to 4 hours, 4 to 12 hours, and 12 to 24 hours after nintedanib administration on day 15 to 16. The urine weight/volume for each collection interval was documented and 2-mL aliquots were stored for bioanalytical measurement.

Plasma and urine concentrations of nintedanib (in the form of its free base BIBF 1120 BS), its metabolite BIBF 1202 (in the form of the free zwitterion BIBF 1202 ZW), and the acylglucuronide thereof (BIBF 1202 glucuronide) were determined by validated assays based on liquid chromatography-tandem mass spectrometry (LC-MS/MS).

For the evaluation of nintedanib plasma protein binding, 10-mL blood samples were collected in devices coated with EDTA before the very first drug administration. *In vitro* protein binding was determined by means of equilibrium dialysis using radio-labelled compound (non–Good Laboratory Practice).

***Statistical Analysis***

The treated set included all patients who received at least one single dose of trial medication and was used for safety and efficacy analyses. The pharmacokinetic set, which was used for all pharmacokinetic analyses, included all evaluable patients in the treated set who provided at least one observation for at least one pharmacokinetic endpoint without important protocol violations relevant to the evaluation of pharmacokinetics. An intent-to-treat approach was used for all analyses although important protocol violations were described. TTP, OS, and PFS were assessed based on the Kaplan-Meier method for each group separately; point estimates, together with CIs (based on the Brookmeyer and Crowley method), were provided for median TTP, median OS, and median PFS. Estimates of the effect of nintedanib versus sorafenib treatment on TTP, OS, and PFS were given by the HR and its 95% CI, using a stratified Cox proportional hazard model.

***Sample Size Determination for Phase II***

Assuming median TTP times of 9 months and 6 months for the nintedanib treatment arm and the sorafenib treatment arm, respectively, we obtain the following probabilities for a correct selection of the nintedanib based on 60 versus 30 patients for the corresponding arms. The trial will be designed to provide a high probability of recording a numerically positive

treatment effect. The probability of observing any numerically positive treatment effect on TTP (i.e., an estimated HR for TTP between nintedanib and sorafenib of less than 1) is around 93%. The probability of observing of 15% reduction in hazard over sorafenib (HR<0.85) will be about 80.9%.

Table. Probability of observing hazard ratio from an exponential distributed survival data (30 vs. 60 patients)

| Underlying true | | | Probability of observing | | | |
| --- | --- | --- | --- | --- | --- | --- |
| TTP  sorafenib | TTP nintedanib | HR | HR<0.8 | HR<0.85 | HR<1 | medC<medT |
| 6 | 9 | 0.67 | 72.9 | 80.9 | 93.4 | 83.5 |
| 6 | 8 | 0.75 | 58.4 | 67.6 | 81.6 | 81.8 |
| 6 | 7 | 0.86 | 39 | 48.7 | 70.6 | 65.8 |
| 6 | 6 | 1 | 22 | 27.2 | 51.8 | 51.6 |

accrual=8 mo, follow-up=14 mo, 30 vs 60 patients, based on 1,000 simulations

Note that these probabilities are only to be regarded from an exploratory viewpoint and cannot be considered as a justification for the chosen sample size. No adjustments for dropouts during the studies have been performed in these calculations.

| Observed PFS events | Observed median TTP (months) | | Observed hazard ratio (nintedanib vs. sorafenib) | Upper limit of two-sided 95% confidence interval (95% CI width) |
| --- | --- | --- | --- | --- |
|  | Nintedanib | Sorafenib |  |  |
| 63 (70%) | 4.5 | 3 | 0.67 | 1.13 (0.73) |
| 72 (80%) | 4.5 | 3 | 0.67 | 1.09 (0.68) |
| 81 (90%) | 4.5 | 3 | 0.67 | 1.06 (0.64) |

The table above shows that, with 90 patients in total and at least 72 event (80% of patients) and an observed hazard ratio of 0.67, the corresponding two-sided 95% confidence interval will cover the positive region of hazard ratio in favour of nintedanib with greater than 87% chance.

**Results**

***Dose-limiting toxicities experienced after the MTD determination period***

Three patients in group I and 7 patients in group II experienced DLTs at various dose levels. For group I, in the 100-mg cohort there were 3 DLTs in 2 patients: two grade 3 bilirubin-increase DLTs and one grade 2 abnormal liver function test; in the 150-mg cohort there was a grade 3 AST-increase DLT. For group II, there were the following DLTs: grade 4 amylase increase in the 50-mg cohort; grade 3 ALP increase and grade 3 hypertension in a patient in the 100-mg cohort; grade 3 amylase increase and grade 4 hyperuricemia in one patient and grade 5 multi-organ failure in another patient in the 150-mg cohort; and for the 200-mg cohort grade 3 DLTs each of bilirubin increase, ascites, and vomiting (the latter two occurring in the same patient); in another patient grade 3 DLTs each of decreased appetite and fatigue as well as a grade 2 drug-induced liver injury and a grade 1 encephalopathy.

***Pharmacokinetics***

Plasma and urine samples of 33 patients were included in the pharmacokinetic evaluation: 18 patients in group I (some of whom were from the phase II portion; 4 patients in the 100-mg bid cohort, 3 patients in the 150-mg bid cohort, and 11 patients in the 200-mg bid cohort), and 15 patients in group II, including patients in the expansion cohort (3 patients in the 50-mg bid cohort, 3 patients in the 100-mg bid cohort, 4 patients in the 150-mg bid cohort, and 5 patients in the 200-mg bid cohort). One patient in the 150-mg bid cohort was categorized as Child-Pugh B; all of the other patients were categorized as Child-Pugh A. Nine patients from the phase II component of the trial were included for PK analysis in group I from the 200-mg bid cohort.

Nintedanib was rapidly absorbed followed by at least biphasic disposition kinetics (supplementary Figure S2). Maximum plasma concentrations were achieved around a median of 2 hours (supplementary Table S4). Trough plasma concentrations maintained a steady state throughout the observation period. BIBF 1202 plasma concentrations reached a maximum around a median of 3 hours and also maintained steady state throughout the observation period. BIBF 1202 glucuronide plasma concentrations reached a plateau and stayed constant over the observation period.

The maximum plasma concentrations (C_max,ss_ values) as well as exposure in terms of AUC_0‑12,ss_ values of nintedanib increased with increasing doses over the dose range tested (supplementary Table S4). High interpatient variability of pharmacokinetic parameters over all dose cohorts was observed. The dose-normalized exposure parameters (C_max,ss,norm_ and AUC_0-12,ss,norm_) generally remained constant over the different dose cohorts, taking differences in patient numbers between dose cohorts and the high variability of pharmacokinetic parameters into account. Renal elimination of nintedanib was low. Twelve hours after repeated dose administration between 0.151% and 0.238% of the administered dose was recovered in urine (fe_0-12,ss_). Renal clearance (CL_R,ss_) of nintedanib was low (13.9 to 16.8 mL/min) and did not change with the dose administered. Exposure to the two major metabolites of nintedanib, BIBF 1202 and BIBF 1202 glucuronide, generally increased in proportion to the nintedanib dose, taking the high variability into account (supplementary Table S4).

No meaningful comparison could be made between patients with HCC in either Child-Pugh category A or B because data for analysis were available for only one patient in Child-Pugh category B (supplementary Table S5).

The overall mean plasma protein binding of nintedanib was comparable between HCC patients and control healthy samples with mean bound fraction (f_B_) values of 98.1 ± 1.1% and 97.4 ± 1.4%, respectively. Corresponding mean unbound fractions (f_U_) were at 1.92 ± 1.12% and 2.67 ± 1.42%, respectively. The mean protein binding between groups I and II was comparable with mean f_U_ ranging from 1.28 to 2.03%. The free fraction observed in samples from patients assigned to Child-Pugh category A was also comparable to those observed in groups I and II, with a mean f_U_ of 1.90 ± 1.11%. The protein unbound fraction in samples from the patient assigned to Child-Pugh category B was 3.61% (sample from 1 patient). The number of samples was limited but the f_U_ value was within the range of variability observed for the other patient subpopulations as well as for the healthy volunteer controls.

***Safety and tolerability***

Phase I

No AEs led to dose reduction in group I and 3/19 (15.8%) patients had AEs leading to dose reduction in group II over the entire treatment period. In group II, grade 4 and grade 3 amylase increase led to dose reduction in patients in the 50-mg and 150-mg cohorts, respectively; in the 200-mg cohort one patient had grade 3 vomiting and grade 2 lower respiratory tract infection that also led to dose reductions. There were 8/13 (61.5%) and 14/19 (73.7%) patients that had AEs leading to treatment discontinuation in group I and group II, respectively.

The most frequent any-grade, any-cause AEs, by system organ class, were gastrointestinal (84.6% for group I, 89.5% for group II) which includes upper/abdominal pain, vomiting, diarrhea, and nausea; and general disorders and administration site conditions (69.2% for group I, 79.0% for group II) which includes fatigue and influenza-like illness. Class-related AEs occurred in both treatment groups and included a case each of grade 1 and grade 3 hypertension and 3 cases of grade 1 rash; there were 2 cases of grade 1 palmar-plantar erythrodysesthesia in group II (1 at 100 mg bid, 1 at 200 mg bid). Supplementary Table S9 shows grade ≥3 AEs of special interest occurring in all the dose cohorts for groups I and II. In total, over both groups there were 15 patients (46.9%) with bleeding and 17 patients (53.1%) with abnormal liver-related investigations. Regarding bleeding of any grade, for group I there was a patient each with bleeding due to hepatic hemorrhage, catheter site bruise, injection site bruising, gastrointestinal hemorrhage, and intra-abdominal hematoma; for group II 15.8% each were due to hematemesis, rectal hemorrhage, and epistaxis (some occurring in the same patient), with the remainder in lesser proportions from bleeding varicose vein, gingival bleeding, hemoptysis, melena, catheter site bruise, injection site bruising, contusion, and hematochezia.

Grade 5 AEs (fatal AEs with onset on treatment or within 28 days of last nintedanib dose) occurred in 8 patients: 1 patient because of cardiac arrest (considered not related to nintedanib) and 1 patient as a result of multi-organ failure (considered possibly related to nintedanib). The remaining 6 patients died of progressive disease, with 2 patients dying because of associated sepsis (1 of which was biliary sepsis).
