# Supplementary figures and images for: A multicentre, open-label, phase-I/randomised phase-II study to evaluate safety, pharmacokinetics, and efficacy of nintedanib vs. sorafenib in European patients with advanced hepatocellular carcinoma
Source: Br J Cancer. 2018 Mar 22;118(9):1162–8. doi: 10.1038/s41416-018-0051-8 (PMC5943284; doi:10.1038/s41416-018-0051-8)

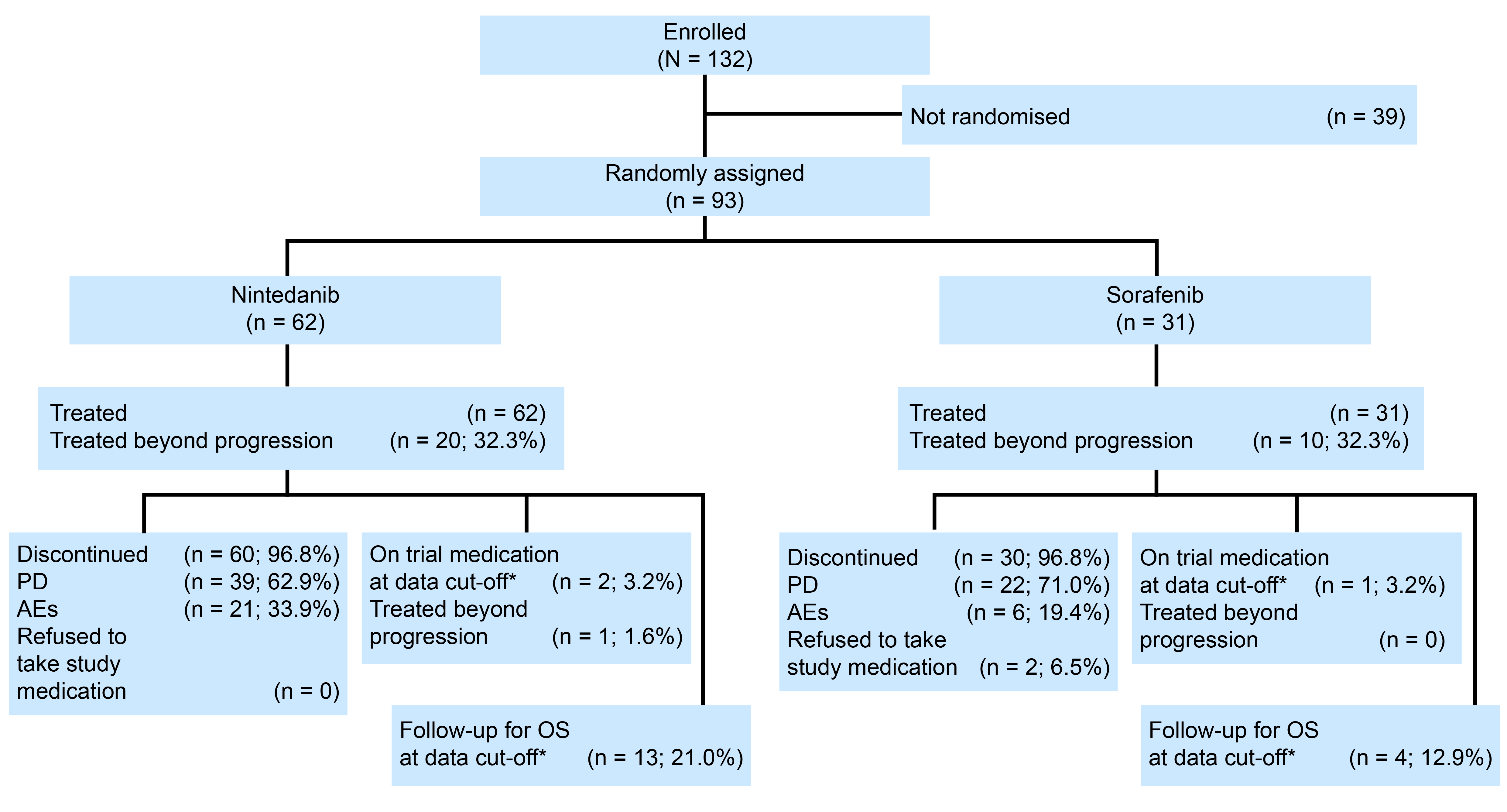

Supplement: Supplementary file 2 — Supplementary Figure S1(TIF 1099 kb) [file 41416_2018_51_MOESM2_ESM.tif]

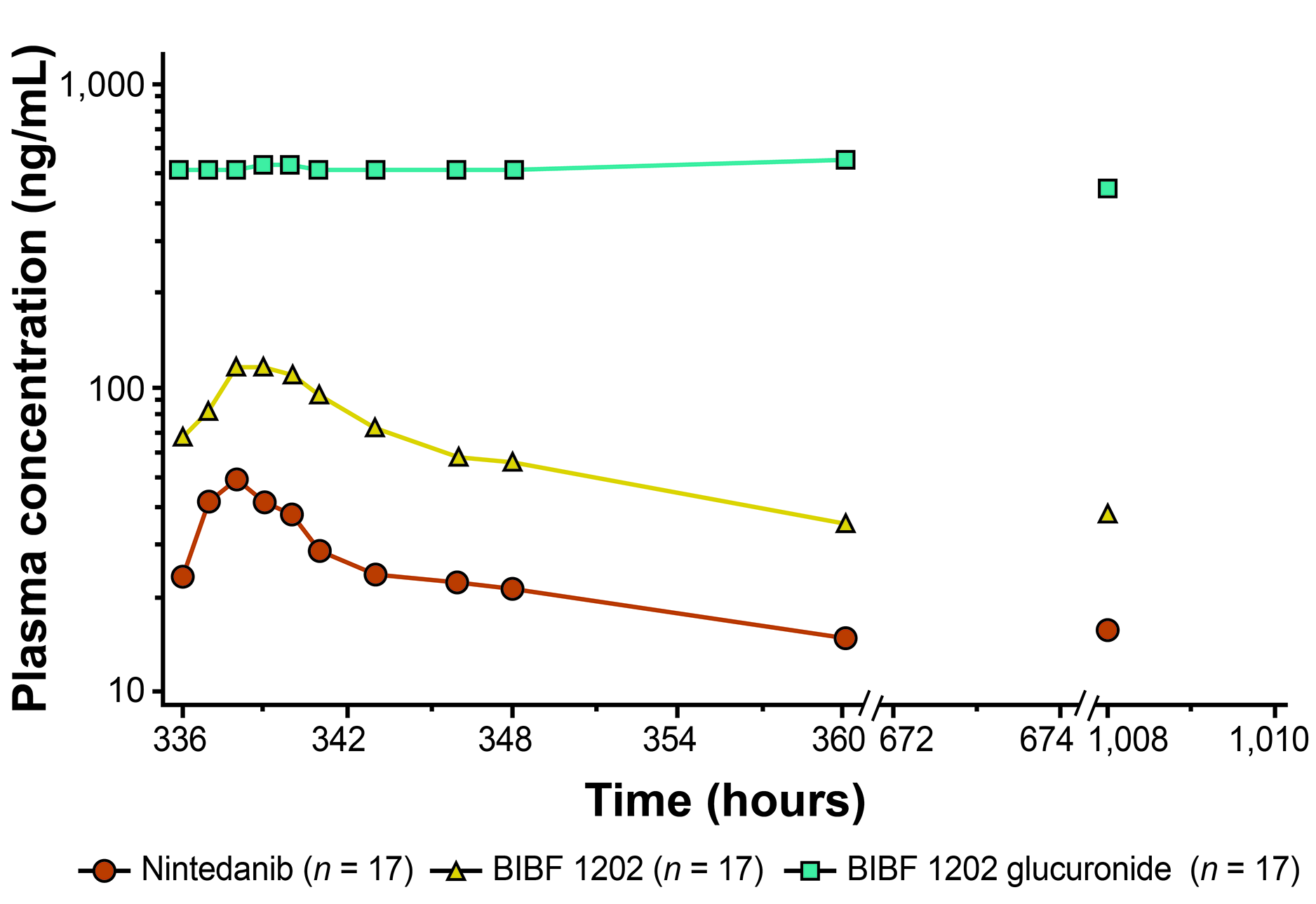

Supplement: Supplementary file 3 — Supplementary Figure S2(TIF 745 kb) [file 41416_2018_51_MOESM3_ESM.tif]

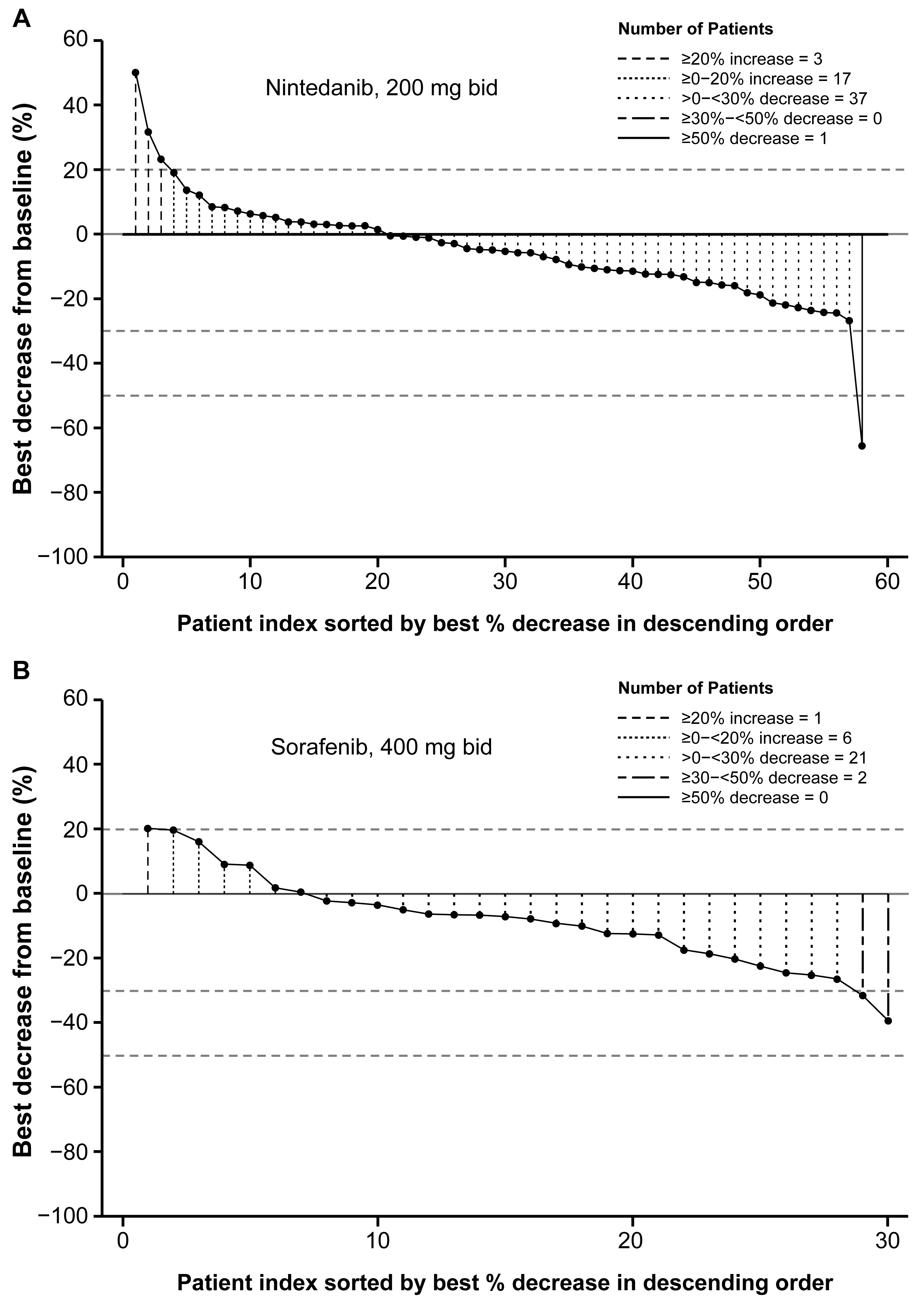

Supplement: Supplementary file 4 — Supplementary Figure S3(TIF 405 kb) [file 41416_2018_51_MOESM4_ESM.tif]
