# Supplementary material for: A multicentre, open-label, phase-I/randomised phase-II study to evaluate safety, pharmacokinetics, and efficacy of nintedanib vs. sorafenib in European patients with advanced hepatocellular carcinoma
Source: Br J Cancer. 2018 Mar 22;118(9):1162–8. doi: 10.1038/s41416-018-0051-8 (PMC5943284; doi:10.1038/s41416-018-0051-8)
Supplement: Supplementary file 5 — Supplementary Table S1(DOCX 33 kb) [file 41416_2018_51_MOESM5_ESM.docx]

| **Supplementary Table S1.** **Phase I patient demographics and baseline disease characteristics** | | | | | | | | | |
| --- | --- | --- | --- | --- | --- | --- | --- | --- | --- |
|  | **Group I** | | | | **Group II** | | | | |
| **Characteristic** | **Nintedanib, 100 mg bid** | **Nintedanib, 150 mg bid** | **Nintedanib, 200 mg bid** | **Total** | **Nintedanib, 50 mg bid** | **Nintedanib, 100 mg bid** | **Nintedanib, 150 mg bid** | **Nintedanib, 200 mg bid** | **Total** |
| Number of patients, *n* | 6 | 3 | 4 | 13 | 3 | 4 | 4 | 8 | 19 |
| Median age, y (range) | 72.0 (56–74) | 61.0 (60–74) | 67.5 (60–74) | 70.0 (56–74) | 70.0 (62–85) | 55.5 (50–64) | 60.0 (42–75) | 62.0 (38–68) | 62.0 (38–85) |
| Gender, *n* (%) |  |  |  |  |  |  |  |  |  |
| Male | 5 (83.3) | 2 (66.7) | 4 (100) | 11 (84.6) | 3 (100) | 4 (100) | 3 (75.0) | 6 (75.0) | 16 (84.2) |
| Female | 1 (16.7) | 1 (33.3) | 0 | 2 (15.4) | 0 | 0 | 1 (25.0) | 2 (25.0) | 3 (15.8) |
| Race, *n* (%) |  |  |  |  |  |  |  |  |  |
| Black | 1 (16.7) | 0 | 0 | 1 (7.7) | 1 (33.3) | 1 (25.0) | 1 (25.0) | 1 (12.5) | 4 (21.1) |
| Caucasian | 5 (83.3) | 3 (100) | 4 (100) | 12 (92.3) | 2 (66.7) | 2 (50.0) | 1 (25.0) | 7 (87.5) | 12 (63.2) |
| Median time since diagnosis, mo (range) | 8.1 (1.0–20.7) | 1.5 (1.1–8.5) | 7.0 (1.0–15.2) | 1.8 (1.0–20.7) | 3.0 (0.8–3.6) | 6.8 (0.7–12.2) | 1.8 (0.5–15.1) | 2.4 (0.2–12.0) | 2.5 (0.2–15.1) |
| ECOG PS, *n* (%) |  |  |  |  |  |  |  |  |  |
| 0 | 5 (83.3) | 3 (100) | 3 (75) | 11 (84.6) | 2 (66.7) | 2 (50.0) | 0 | 4 (50.0) | 8 (42.1) |
| 1 | 1 (16.7) | 0 | 1 (25.0) | 2 (15.4) | 1 (33.3) | 2 (50.0) | 3 (75.0) | 4 (50.0) | 10 (52.6) |
| 2 | 0 | 0 | 0 | 0 | 0 | 0 | 1 (25.0) | 0 | 1 (5.3) |
| Child-Pugh score, *n* (%) |  |  |  |  |  |  |  |  |  |
| 5 | 4 (66.7) | 3 (100) | 3 (75.0) | 10 (76.9) | 3 (100) | 3 (75.0) | 2 (50.0) | 2 (25.0) | 10 (52.6) |
| 6 | 2 (33.3) | 0 | 1 (25.0) | 3 (23.1) | 0 | 0 | 1 (25.0) | 6 (75.0) | 7 (36.8) |
| 7 | 0 | 0 | 0 | 0 | 0 | 1 (25.0) | 1 (25.0) | 0 | 2 (10.5) |
| BCLC stage, *n* (%) |  |  |  |  |  |  |  |  |  |
| 0 | 0 | 0 | 0 | 0 | 0 | 0 | 0 | 0 | 0 |
| A | 0 | 0 | 0 | 0 | 0 | 0 | 0 | 0 | 0 |
| B | 4 (66.7) | 0 | 1 (25.0) | 5 (38.5) | 0 | 1 (25.0) | 0 | 1 (12.5) | 2 (10.5) |
| C | 2 (33.3) | 3 (100) | 3 (75.0) | 8 (61.5) | 3 (100) | 3 (75.0) | 4 (100) | 7 (87.5) | 17 (89.5) |
| D | 0 | 0 | 0 | 0 | 0 | 0 | 0 | 0 | 0 |
| MVI present, *n* (%) | 2 (33.3) | 1 (33.3) | 1 (25.0) | 4 (30.8) | 1 (33.3) | 1 (25.0) | 2 (50.0) | 6 (75.0) | 10 (52.6) |
| EHS present, *n* (%) | 3 (50.) | 3 (100) | 2 (50.0) | 8 (61.5) | 3 (100) | 2 (50.0) | 2 (50.0) | 2 (25.0) | 9 (47.4) |
| Location of EHS, *n* (%) |  |  |  |  |  |  |  |  |  |
| Bone | 0 | 0 | 1 (25.0) | 1 (7.7) | 0 | 1 (25.0) | 0 | 0 | 1 (5.3) |
| Lung | 1 (16.7) | 0 | 1 (25.0) | 2 (15.4) | 0 | 2 (50.0) | 2 (50.0) | 1 (12.5) | 5 (26.3) |
| Lymph | 3 (50.0) | 2 (66.7) | 1 (25.0) | 6 (46.2) | 3 (100) | 1 (25.0) | 0 | 1 (12.5) | 5 (26.3) |
| Other | 0 | 2 (66.7) | 0 | 2 (15.4) | 2 (66.7) | 0 | 1 (25.0) | 1 (12.5) | 4 (21.1) |
| Aetiology of parenchymal liver disease, *n* (%) |  |  |  |  |  |  |  |  |  |
| Alcohol  Related | 3 (50.0) | 0 | 2 (50.0) | 5 (38.5) | 0 | 0 | 0 | 2 (25.0) | 2 (10.5) |
| HBV related | 0 | 0 | 0 | 0 | 2 (66.7) | 0 | 1 (25.0) | 1 (12.5) | 4 (21.1) |
| HCV related | 1 (16.7) | 0 | 0 | 1 (7.7) | 0 | 2 (50.0) | 2 (50.0) | 3 (37.5) | 7 (36.8) |
| HBV + HCV  Related | 0 | 0 | 0 | 0 | 0 | 0 | 0 | 0 | 0 |
| Unknown | 2 (33.3) | 0 | 0 | 2 (15.4) | 1 (33.3) | 2 (50.0) | 0 | 1 (12.5) | 4 (21.1) |
| Other | 0 | 3 (100) | 2 (50.0) | 5 (38.5) | 0 | 0 | 1 (25.0) | 1 (12.5) | 2 (10.5) |
| Parenchymal liver disease, *n* (%) |  |  |  |  |  |  |  |  |  |
| Chronic  hepatitis | 0 | 0 | 0 | 0 | 0 | 1 (25.0) | 0 | 0 | 1 (5.3) |
| Steatofibrosis | 1 (16.7) | 0 | 0 | 1 (7.7) | 0 | 0 | 0 | 0 | 0 |
| Cirrhosis | 5 (83.3) | 0 | 2 (50.0) | 7 (53.8) | 2 (66.7) | 2 (50.0) | 2 (50.0) | 6 (75.0) | 12 (63.2) |
| No evidence | 0 | 3 (100) | 1 (25.0) | 4 (30.8) | 1 (33.3) | 1 (25.0) | 1 (25.0) | 1 (12.5) | 4 (21.1) |
| Unknown | 0 | 0 | 0 | 0 | 0 | 0 | 1 (25.0) | 0 | 1 (5.3) |
| Other | 0 | 0 | 1 (25.0) | 1 (7.7) | 0 | 0 | 0 | 1 (12.5) | 1 (5.3) |
| Type of local therapy, *n* (%) |  |  |  |  |  |  |  |  |  |
| Complete  surgical  resection | 0 | 0 | 0 | 0 | 0 | 0 | 0 | 0 | 0 |
| RFA | 0 | 0 | 1 (25.0) | 1 (7.7) | 0 | 0 | 0 | 0 | 0 |
| PEI | 0 | 0 | 0 | 0 | 0 | 0 | 0 | 0 | 0 |
| TACE | 0 | 0 | 0 | 0 | 0 | 0 | 0 | 2 (25.0) | 2 (10.5) |
| RT | 0 | 0 | 0 | 0 | 0 | 0 | 0 | 1 (12.5) | 1 (5.3) |
| Other | 0 | 0 | 0 | 0 | 0 | 0 | 1 (25.0) | 0 | 1 (5.3) |
| Abbreviations: BCLC, Barcelona Clinic Liver Cancer; ECOG PS, Eastern Cooperative Oncology Group performance status; EHS, extrahepatic spread; HBV, hepatitis B; HCV, hepatitis C; MVI, macrovascular invasion; PEI, percutaneous ethanol injection; RFA, radiofrequency ablation; RT, radiotherapy; TACE, transarterial chemoembolization. | | | | | | | | | |
