# Supplementary material for: A multicentre, open-label, phase-I/randomised phase-II study to evaluate safety, pharmacokinetics, and efficacy of nintedanib vs. sorafenib in European patients with advanced hepatocellular carcinoma
Source: Br J Cancer. 2018 Mar 22;118(9):1162–8. doi: 10.1038/s41416-018-0051-8 (PMC5943284; doi:10.1038/s41416-018-0051-8)
Supplement: Supplementary file 6 — Supplementary Table S2(DOCX 25 kb) [file 41416_2018_51_MOESM6_ESM.docx]

| **Supplementary Table S2.** **Phase I patient disposition** | | | | | | | | | |
| --- | --- | --- | --- | --- | --- | --- | --- | --- | --- |
|  | **Group I** | | | | **Group II** | | | | |
| **Parameter, *n* (%)** | **Nintedanib, 100 mg bid** | **Nintedanib, 150 mg bid** | **Nintedanib, 200 mg bid** | **Total** | **Nintedanib, 50 mg bid** | **Nintedanib, 100 mg bid** | **Nintedanib, 150 mg bid** | **Nintedanib, 200 mg bid** | **Total** |
| Discontinued from trial medication | 6 (100) | 3 (100) | 4 (100) | 13 (100) | 3 (100) | 4 (100) | 4 (100) | 8 (100) | 19 (100) |
| Progressive  disease | 2 (33.3) | 1 (33.3) | 1 (25.0) | 4 (30.8) | 0 | 2 (50.0) | 1 (25.0) | 1 (12.5) | 4 (21.1) |
| AEs | 4 (66.7) | 2 (66.7) | 3 (75.0) | 9 (69.2) | 3 (100) | 2 (50) | 2 (50) | 7 (87.5) | 14 (73.7) |
| DLT | 2 (33.3) | 1 (33.3) | 0 | 3 (23.1) | 0 | 1 (25.0) | 0 | 2 (25.0) | 3 (15.8) |
| Other AE | 2 (33.3) | 1 (33.3) | 3 (75.0) | 6 (46.2) | 3 (100) | 1 (25.0) | 2 (50.0) | 5 (62.5) | 11 (57.9) |
| Non-  compliant  with protocol | 0 | 0 | 0 | 0 | 0 | 0 | 0 | 0 | 0 |
| Lost to  follow-up | 0 | 0 | 0 | 0 | 0 | 0 | 0 | 0 | 0 |
| Refused to  continue  taking trial  medication | 0 | 0 | 0 | 0 | 0 | 0 | 0 | 0 | 0 |
| Other | 0 | 0 | 0 | 0 | 0 | 0 | 1 (25.0) | 0 | 1 (5.3) |
| Abbreviations: AE, adverse events; DLT, dose-limiting toxicity | | | | | | | | | |
