# Supplementary material for: A multicentre, open-label, phase-I/randomised phase-II study to evaluate safety, pharmacokinetics, and efficacy of nintedanib vs. sorafenib in European patients with advanced hepatocellular carcinoma
Source: Br J Cancer. 2018 Mar 22;118(9):1162–8. doi: 10.1038/s41416-018-0051-8 (PMC5943284; doi:10.1038/s41416-018-0051-8)
Supplement: Supplementary file 7 — Supplementary Table S3(DOCX 25 kb) [file 41416_2018_51_MOESM7_ESM.docx]

| **Supplementary Table S3. Phase I nintedanib dose reductions and exposure** | | | | | | | |
| --- | --- | --- | --- | --- | --- | --- | --- |
|  | **Group I** | | | **Group II** | | | |
|  | **Nintedanib, 100 mg bid** | **Nintedanib, 150 mg bid** | **Nintedanib, 200 mg bid** | **Nintedanib, 50 mg bid** | **Nintedanib, 100 mg bid** | **Nintedanib, 150 mg bid** | **Nintedanib, 200 mg bid** |
| Number of dose reductions, *n* (%) |  |  |  |  |  |  |  |
| 0 | 6 (100) | 3 (100) | 2 (50.0) | 3 (100) | 4 (100) | 3 (75.0) | 6 (75.0) |
| 1 | 0 | 0 | 2 (50.0) | 0 | 0 | 1 (25.0) | 0 |
| 2 | 0 | 0 | 0 | 0 | 0 | 0 | 2 (25.0) |
| Median time to first dose reduction, days (range) | - | - | 306.5 (283–330) | - | - | 15.0 | 67.5 (32­–103) |
| Median dose intensity | 100% | 100% | 99.3% | 100% | 100% | 100% | 100% |
| Median duration of treatment, days (range) | 214 (30–785) | 241 (226–483) | 559 (43–1,261) | 130 (93–456) | 68.5 (42–113) | 100.5 (20–336) | 74.5 (27–365) |
