# Supplementary material for: A multicentre, open-label, phase-I/randomised phase-II study to evaluate safety, pharmacokinetics, and efficacy of nintedanib vs. sorafenib in European patients with advanced hepatocellular carcinoma
Source: Br J Cancer. 2018 Mar 22;118(9):1162–8. doi: 10.1038/s41416-018-0051-8 (PMC5943284; doi:10.1038/s41416-018-0051-8)
Supplement: Supplementary file 8 — Supplementary Table S4(DOCX 29 kb) [file 41416_2018_51_MOESM8_ESM.docx]

| **Supplementary Table S4.** **Geometric mean (and gCV%) or individual pharmacokinetic parameters of nintedanib, BIBF 1202, and BIBF 1202 glucuronide after multiple oral administration of nintedanib by dose cohort for phase I and phase II** | | | | |
| --- | --- | --- | --- | --- |
|  | **Dose of Nintedanib** | | | |
| **PK parameter (units)** | **50 mg bid** | **100 mg bid** | **150 mg bid** | **200 mg bid** |
|  | **gMean (gCV%)** | | | |
|  | **Nintedanib** | | | |
| C_max,ss_ (nmol/L) | 12.9 (12.1)  *n* = 3 | 38.1 (58.6)  *n* = 7 | 72.2 (105)  *n* = 6 | 69.1 (118)  *n* = 17 |
| C_max,ss,norm_ ([nmol/L]/mg) | 0.259 (12.1)  *n* = 3 | 0.381 (58.6)  *n* = 7 | 0.481 (105)  *n* = 6 | 0.346 (118)  *n* = 17 |
| t_max,ss_^a^ (h) | 1.05 (0.950 to 3.00)  *n* = 3 | 2.98 (0.917 to 3.97)  *n* = 7 | 2.03 (1.30 to 4.00)  *n* = 6 | 2.02 (0 to 10.0)  *n* = 17 |
| t_1/2,ss_ (h) | 33.0 (65.2)  *n* = 3 | 19.7 (35.2)  *n* = 7 | 18.3 (34.8)  *n* = 6 | 20.1 (85.5)  *n* = 17 |
| AUC_0-12,ss_ ([nmol•h]/L) | 75.0 (17.7)  *n* = 3 | 228 (53.3)  *n* = 7 | 390 (67.6)  *n* = 6 | 390 (103)  *n* = 17 |
| AUC_0-12,ss,norm_ (nmol•h/L/mg) | 1.50 (17.7)  *n* = 3 | 2.28 (53.3)  *n* = 7 | 2.60 (67.6)  *n* = 6 | 1.95 (103)  *n* = 17 |
| Ae_0-12,ss_ (µg) | 75.5 (46.4)  *n* = 3 | 212 (99.7)  *n* = 7 | 357 (48.7)  *n* = 6 | 348 (108)  *n* = 16 |
| fe_0-12,ss_ (%) | 0.151 (46.4)  *n* = 3 | 0.212 (99.7)  *n* = 7 | 0.238 (48.7)  *n* = 6 | 0.185 (108)  *n* = 15 |
| CL_R,ss_ (mL/min) | 16.8 (34.1)  *n* = 3 | 15.5 (57.9)  *n* = 7 | 15.3 (27.5)  *n* = 6 | 13.9 (53.7)  *n* = 16 |
|  | **BIBF 1202** | | | |
| C_max,ss,norm_ ([nmol/L]/mg) | 0.469 (39.8)  *n* = 3 | 0.565 (142)  *n* = 7 | 0.578 (117)  *n* = 6 | 0.713 (219)  *n* = 17 |
| t_max,ss_^a^ (h) | 2.00 (0 to 3.00)  *n* = 3 | 3.98 (1.98 to 5.08)  *n* = 7 | 2.63 (0 to 5.03)  *n* = 6 | 2.98 (0 to 11.0)  *n* = 17 |
| AUC_0-12,ss,norm_ (nmol•h/L/mg) | 3.97 (43.0)  *n* = 3 | 4.61 (164)  *n* = 7 | 4.39 (127)  *n* = 6 | 5.11 (227)  *n* = 17 |
|  | **BIBF 1202 glucuronide** | | | |
| C_max,ss,norm_ ([nmol/L]/mg) | 1.51 (159)  *n* = 3 | 2.99 (176)  *n* = 7 | 1.87 (71.1)  *n* = 6 | 3.00 (194)  *n* = 17 |
| t_max,ss_^a^ (h) | 3.00 (2.00 to 10.0)  *n* = 3 | 4.08 (0 to 11.0)  *n* = 7 | 3.61 (0 to 11.0)  *n* = 6 | 3.00 (0 to 11.0)  *n* = 17 |
| AUC_0-12,ss,norm_ (nmol•h/L/mg) | 15.9 (147)  *n* = 3 | 31.6 (175)  *n* = 7 | 20.1 (69.5)  *n* = 6 | 36.7 (167)  *n* = 16 |
| Abbreviations: Ae_0-12,ss_, amount of analyte that is eliminated in urine at steady state over the time interval 0 to 12 hours; CLR_,ss_, renal clearance of the analyte at steady state based on 0–12-hour data; fe_0-12,ss_, fraction of analyte excreted unchanged in urine at steady state over the time interval 0 to 12 hours; gCV, geometric mean of the coefficient of variation; PK, pharmacokinetic.  ^a^Median and range | | | | |
