# Supplementary material for: A multicentre, open-label, phase-I/randomised phase-II study to evaluate safety, pharmacokinetics, and efficacy of nintedanib vs. sorafenib in European patients with advanced hepatocellular carcinoma
Source: Br J Cancer. 2018 Mar 22;118(9):1162–8. doi: 10.1038/s41416-018-0051-8 (PMC5943284; doi:10.1038/s41416-018-0051-8)
Supplement: Supplementary file 9 — Supplementary Table S5(DOCX 27 kb) [file 41416_2018_51_MOESM9_ESM.docx]

| **Supplementary Table S5. Geometric mean (and gCV%) key dose-normalized pharmacokinetic parameters of nintedanib and its metabolites after multiple oral administration of nintedanib in group I and group II, and patients with Child-Pugh category A and B, for phase I and phase II** | | | | | | |
| --- | --- | --- | --- | --- | --- | --- |
|  | **Group I** | **Group II** | **gMean ratio** | **Child-Pugh A** | **Child-Pugh B^a^** | **gMean ratio** |
|  | **gMean (gCV%)** | **gMean (gCV%)** |  | **gMean (gCV%)** | **Value** |  |
|  | **Nintedanib** | | | | | |
| AUC_0-12,ss,norm_ ([ng·h/mL]/mg) | 1.73 (78.8)  *n* = 19 | 2.64 (73.6)  *n* = 14 | 1.53 | 2.10 (80.8)  *n* = 32 | 1.37 | NC |
| C_max,ss,norm_ ([g/mL]/mg) | 0.326 (101)  *n* = 19 | 0.426 (85.3)  *n* = 14 | 1.31 | 0.374 (94.1)  *n* = 32 | 0.163 | NC |
| fe_0-12,ss_ (%) | 0.154 (73.5)  *n* = 18 | 0.276 (88.5)  *n* = 13 | 1.79 | 0.198 (88.7)  *n* = 30 | 0.156 | NC |
|  | **BIBF 1202** | | | | | |
| AUC_0-12,ss,norm_ ([ng·h/mL]/mg) | 3.27 (180)  *n* = 19 | 7.90 (107)  *n* = 14 | 2.42 | 4.71 (170)  *n* = 32 | 6.50 | NC |
| C_max,ss,norm_ ([ng/mL]/mg) | 0.469 (186)  *n* = 19 | 0.936 (102)  *n* = 14 | 2.00 | 0.626 (162)  *n* = 32 | 0.733 | NC |
|  | **BIBF 1202 glucuronide** | | | | | |
| AUC_0-12,ss,norm_ ([ng·h/mL]/mg) | 25.2 (135)  *n* = 18 | 35.6 (163)  *n* = 14 | 1.41 | 29.1 (150)  *n* = 31 | 35.8 | NC |
| C_max,ss,norm_ ([ng/mL]/mg) | 2.14 (150)  *n* = 19 | 3.33 (166)  *n* = 14 | 1.56 | 2.56 (162)  *n* = 32 | 3.31 | NC |
| Abbreviations: fe_0-12,ss_, fraction of analyte excreted unchanged in urine at steady state over the time interval 0 to 12 hours; gCV, geometric mean of the coefficient of variation; NC, not calculated  ^a^There was only one patient with Child-Pugh category B. | | | | | | |
