# Supplementary material for: A multicentre, open-label, phase-I/randomised phase-II study to evaluate safety, pharmacokinetics, and efficacy of nintedanib vs. sorafenib in European patients with advanced hepatocellular carcinoma
Source: Br J Cancer. 2018 Mar 22;118(9):1162–8. doi: 10.1038/s41416-018-0051-8 (PMC5943284; doi:10.1038/s41416-018-0051-8)
Supplement: Supplementary file 10 — Supplementary Table S6(DOCX 26 kb) [file 41416_2018_51_MOESM10_ESM.docx]

| **Supplementary Table S6.** **Phase II objective radiological response according to RECIST and mRECIST by central independent review** | | |
| --- | --- | --- |
| Best Response | Treatment Group | |
|  | Nintedanib, 200 mg bid (n = 62) | Sorafenib, 400 mg bid (n = 31) |
| RECIST, n (%) | | |
| Disease control | 51 (82.3) | 28 (90.3) |
| Objective  response | 1 (1.6) | 2 (6.5) |
| Complete  response | 0 (0.0) | 0 (0.0) |
| Partial  response | 1 (1.6) | 2 (6.5) |
| Stable disease | 50 (80.6) | 26 (83.9) |
| Progressive disease | 8 (12.9) | 1 (3.2) |
| Not evaluable | 2 (3.2) | 0 (0.0) |
| Unknown | 1 (1.6) | 2 (6.5) |
| mRECIST, n (%) | | |
| Disease control | 51 (82.3) | 28 (90.3) |
| Objective  response | 7 (11.3) | 6 (19.4) |
| Complete  response | 0 (0.0) | 0 (0.0) |
| Partial  response | 7 (11.3) | 6 (19.4) |
| Stable disease | 44 (71.0) | 22 (71.0) |
| Progressive disease | 8 (12.9) | 1 (3.2) |
| Not evaluable | 2 (3.2) | 0 (0.0) |
| Unknown | 1 (1.6) | 2 (6.5) |
| Abbreviations: RECIST, Response Evaluation Criteria in Solid Tumours; mRECIST, modified RECIST | | |
