# Supplementary material for: A multicentre, open-label, phase-I/randomised phase-II study to evaluate safety, pharmacokinetics, and efficacy of nintedanib vs. sorafenib in European patients with advanced hepatocellular carcinoma
Source: Br J Cancer. 2018 Mar 22;118(9):1162–8. doi: 10.1038/s41416-018-0051-8 (PMC5943284; doi:10.1038/s41416-018-0051-8)
Supplement: Supplementary file 11 — Supplementary Table S7(DOCX 26 kb) [file 41416_2018_51_MOESM11_ESM.docx]

| **Supplementary Table S7.** **Summary of adverse events (CTCAE v3.0) during the phase II portion** | | |
| --- | --- | --- |
|  | **Treatment Group** | |
| Characteristic | Nintedanib, 200 mg bid  (n = 62), n (%) | Sorafenib, 400 mg bid  (n = 31), n (%) |
| Patients with any AE | 62 (100) | 31 (100.0) |
| Patients with investigator defined drug-related AEs | 54 (87.1) | 30 (96.8) |
| Patients with AEs leading to dose reduction of trial drug | 12 (19.4) | 13 (41.9) |
| Patients with AEs leading to discontinuation of trial drug | 28 (45.2) | 7 (22.6) |
| Patients with serious AEs | 34 (54.8) | 14 (45.2) |
| Fatal | 9 (14.5) | 3 (9.7) |
| Immediately life-threatening | 2 (3.2) | 0 (0.0) |
| Disability/incapacitating | 0 (0.0) | 1 (3.2) |
| Required hospitalization | 31 (50.0) | 14 (45.2) |
| Prolonged hospitalization | 1 (1.6) | 0 (0.0) |
| Congenital anomaly | 0 (0.0) | 0 (0.0) |
| Other | 1 (1.6) | 0 (0.0) |
| Worst CTCAE grade of AEs |  |  |
| Grade 1 | 8 (12.9) | 1 (3.2) |
| Grade 2 | 12 (19.4) | 2 (6.5) |
| Grade 3 | 26 (41.9) | 24 (77.4) |
| Grade 4 | 7 (11.3) | 1 (3.2) |
| Grade 5 | 9 (14.5) | 3 (9.7) |
| Abbreviations: AE, adverse event; CTCAE, Common Terminology Criteria for Adverse Events.  NOTE. A patient may be counted in more than one seriousness criterion. Percentages are calculated using total number of patients per treatment as the denominator. MedDRA v17.0 was used for reporting. | | |
