# Supplementary material for: A multicentre, open-label, phase-I/randomised phase-II study to evaluate safety, pharmacokinetics, and efficacy of nintedanib vs. sorafenib in European patients with advanced hepatocellular carcinoma
Source: Br J Cancer. 2018 Mar 22;118(9):1162–8. doi: 10.1038/s41416-018-0051-8 (PMC5943284; doi:10.1038/s41416-018-0051-8)
Supplement: Supplementary file 12 — Supplementary Table S8(DOCX 47 kb) [file 41416_2018_51_MOESM12_ESM.docx]

| **Supplementary Table S8.** **Adverse events leading to dose reduction and drug discontinuation by CTCAE grade during the phase II portion** | | | | | |
| --- | --- | --- | --- | --- | --- |
|  | **Adverse Event Grade by Treatment Group** | | | | |
| **Adverse Event** | Nintedanib, 200 mg bid  (n = 62) | | Sorafenib, 400 mg bid  (n = 31) | | |
| AEs Leading to Dose Reduction | All grades, n (%) | Grade ≥3, n (%) | All grades, n (%) | Grade ≥3, n (%) | |
| Patients with AEs leading to dose reduction of trial drug | 12 (19.4) | 9 (14.5) | 13 (41.9) | 12 (38.7) |  |
| Blood and lymphatic system disorders | 0 (0.0) | 0 (0.0) | 2 (6.5) | 2 (6.5) |  |
| Neutropenia | 0 (0.0) | 0 (0.0) | 1 (3.2) | 1 (3.2) |  |
| Thrombocytopenia | 0 (0.0) | 0 (0.0) | 1 (3.2) | 1 (3.2) |  |
| Gastrointestinal disorders | 4 (6.5) | 2 (3.2) | 1 (3.2) | 1 (3.2) |  |
| Diarrhoea | 2 (3.2) | 1 (1.6) | 1 (3.2) | 1 (3.2) |  |
| Gastric ulcer | 1 (1.6) | 1 (1.6) | 0 (0.0) | 0 (0.0) |  |
| Nausea | 1 (1.6) | 0 (0.0) | 0 (0.0) | 0 (0.0) |  |
| General disorders and administration site conditions | 2 (3.2) | 1 (1.6) | 1 (3.2) | 1 (3.2) |  |
| Fatigue | 2 (3.2) | 1 (1.6) | 0 (0.0) | 0 (0.0) |  |
| General physical health  deterioration | 0 (0.0) | 0 (0.0) | 1 (3.2) | 1 (3.2) |  |
| Investigations | 5 (8.1) | 5 (8.1) | 1 (3.2) | 1 (3.2) |  |
| ALT increased | 1 (1.6) | 1 (1.6) | 1 (3.2) | 1 (3.2) |  |
| AST increased | 2 (3.2) | 2 (3.2) | 1 (3.2) | 0 (0.0) |  |
| Amylase increased | 1 (1.6) | 1 (1.6) | 0 (0.0) | 0 (0.0) |  |
| Blood alkaline phosphatase  increased | 1 (1.6) | 0 (0.0) | 0 (0.0) | 0 (0.0) |  |
| Blood bilirubin increased | 1 (1.6) | 1 (1.6) | 0 (0.0) | 0 (0.0) |  |
| Lipase increased | 1 (1.6) | 1 (1.6) | 0 (0.0) | 0 (0.0) |  |
| Nervous system disorders | 0 (0.0) | 0 (0.0) | 3 (9.7) | 2 (6.5) |  |
| Hepatic encephalopathy | 0 (0.0) | 0 (0.0) | 1 (3.2) | 1 (3.2) |  |
| Lethargy | 0 (0.0) | 0 (0.0) | 2 (6.5) | 1 (3.2) |  |
| Skin and subcutaneous tissue disorders | 0 (0.0) | 0 (0.0) | 9 (29.0) | 7 (22.6) |  |
| Palmar-plantar  erythrodysesthesia  syndrome | 0 (0.0) | 0 (0.0) | 6 (19.4) | 5 (16.1) |  |
| Rash | 0 (0.0) | 0 (0.0) | 1 (3.2) | 0 (0.0) |  |
| Skin reaction | 0 (0.0) | 0 (0.0) | 2 (6.5) | 2 (6.5) |  |
| Vascular disorders | 1 (1.6) | 1 (1.6) | 0 (0.0) | 0 (0.0) |  |
| Essential hypertension | 1 (1.6) | 1 (1.6) | 0 (0.0) | 0 (0.0) |  |
| Hypertension | 1 (1.6) | 1 (1.6) | 0 (0.0) | 0 (0.0) |  |
| Patients with AEs leading to discontinuation of trial drug | 28 (45.2) | 18 (29.0) | 7 (22.6) | 5 (16.1) |  |
| Blood and lymphatic system disorders | 1 (1.6) | 1 (1.6) | 1 (3.2) | 1 (3.2) |  |
| Anaemia | 1 (1.6) | 1 (1.6) | 1 (3.2) | 1 (3.2) |  |
| Cardiac disorders | 1 (1.6) | 0 (0.0) | 0 (0.0) | 0 (0.0) |  |
| Tachycardia | 1 (1.6) | 0 (0.0) | 0 (0.0) | 0 (0.0) |  |
| Ear and labyrinth disorders | 1 (1.6) | 0 (0.0) | 0 (0.0) | 0 (0.0) |  |
| Vertigo | 1 (1.6) | 0 (0.0) | 0 (0.0) | 0 (0.0) |  |
| Gastrointestinal disorders | 10 (16.1) | 6 (9.7) | 1 (3.2) | 1 (3.2) |  |
| Upper gastrointestinal  haemorrhage | 2 (3.2) | 1 (1.6) | 0 (0.0) | 0 (0.0) |  |
| Vomiting | 2 (3.2) | 1 (1.6) | 0 (0.0) | 0 (0.0) |  |
| Abdominal pain upper | 1 (1.6) | 0 (0.0) | 0 (0.0) | 0 (0.0) |  |
| Ascites | 1 (1.6) | 0 (0.0) | 0 (0.0) | 0 (0.0) |  |
| Diarrhoea | 1 (1.6) | 1 (1.6) | 0 (0.0) | 0 (0.0) |  |
| Gastric varices haemorrhage | 1 (1.6) | 1 (1.6) | 0 (0.0) | 0 (0.0) |  |
| Nausea | 1 (1.6) | 1 (1.6) | 0 (0.0) | 0 (0.0) |  |
| Oesophageal varices  haemorrhage | 1 (1.6) | 1 (1.6) | 0 (0.0) | 0 (0.0) |  |
| Varices oesophageal | 1 (1.6) | 1 (1.6) | 0 (0.0) | 0 (0.0) |  |
| Mouth ulceration | 0 (0.0) | 0 (0.0) | 1 (3.2) | 1 (3.2) |  |
| General disorders and administration site conditions | 9 (14.5) | 8 (12.9) | 0 (0.0) | 0 (0.0) |  |
| Fatigue | 4 (6.5) | 4 (6.5) | 0 (0.0) | 0 (0.0) |  |
| General physical health  deterioration | 3 (4.8) | 2 (3.2) | 0 (0.0) | 0 (0.0) |  |
| Asthenia | 1 (1.6) | 1 (1.6) | 0 (0.0) | 0 (0.0) |  |
| Disease progression | 1 (1.6) | 1 (1.6) | 0 (0.0) | 0 (0.0) |  |
| Performance status decreased | 1 (1.6) | 1 (1.6) | 0 (0.0) | 0 (0.0) |  |
| Hepatobiliary disorders | 1 (1.6) | 1 (1.6) | 1 (3.2) | 0 (0.0) |  |
| Hyperbilirubinemia | 1 (1.6) | 1 (1.6) | 0 (0.0) | 0 (0.0) |  |
| Hepatotoxicity | 0 (0.0) | 0 (0.0) | 1 (3.2) | 0 (0.0) |  |
| Infections and infestations | 1 (1.6) | 0 (0.0) | 0 (0.0) | 0 (0.0) |  |
| Sepsis | 1 (1.6) | 0 (0.0) | 0 (0.0) | 0 (0.0) |  |
| Investigations | 3 (4.8) | 1 (1.6) | 2 (6.5) | 1 (3.2) |  |
| Blood alkaline phosphatase  increased | 2 (3.2) | 0 (0.0) | 0 (0.0) | 0 (0.0) |  |
| Blood bilirubin increased | 2 (3.2) | 1 (1.6) | 0 (0.0) | 0 (0.0) |  |
| ALT increased | 1 (1.6) | 0 (0.0) | 0 (0.0) | 0 (0.0) |  |
| Hepatic enzyme increased | 1 (1.6) | 1 (1.6) | 0 (0.0) | 0 (0.0) |  |
| Electrocardiogram QT  prolongation | 0 (0.0) | 0 (0.0) | 1 (3.2) | 0 (0.0) |  |
| Transaminases increased | 0 (0.0) | 0 (0.0) | 1 (3.2) | 1 (3.2) |  |
| Neoplasms benign, malignant,  and unspecified (including cysts  and polyps) | 1 (1.6) | 1 (1.6) | 0 (0.0) | 0 (0.0) |  |
| Malignant neoplasm  progression | 1 (1.6) | 1 (1.6) | 0 (0.0) | 0 (0.0) |  |
| Nervous system disorders | 2 (3.2) | 1 (1.6) | 0 (0.0) | 0 (0.0) |  |
| Hepatic encephalopathy | 1 (1.6) | 1 (1.6) | 0 (0.0) | 0 (0.0) |  |
| Lethargy | 1 (1.6) | 0 (0.0) | 0 (0.0) | 0 (0.0) |  |
| Psychiatric disorders | 1 (1.6) | 0 (0.0) | 0 (0.0) | 0 (0.0) |  |
| Depressed mood | 1 (1.6) | 0 (0.0) | 0 (0.0) | 0 (0.0) |  |
| Renal and urinary disorders | 1 (1.6) | 0 (0.0) | 0 (0.0) | 0 (0.0) |  |
| Nephrotic syndrome | 1 (1.6) | 0 (0.0) | 0 (0.0) | 0 (0.0) |  |
| Skin and subcutaneous tissue  disorders | 0 (0.0) | 0 (0.0) | 4 (12.9) | 3 (9.7) |  |
| Palmar-plantar  erythrodysesthesia  syndrome | 0 (0.0) | 0 (0.0) | 2 (6.5) | 1 (3.2) |  |
| Rash | 0 (0.0) | 0 (0.0) | 1 (3.2) | 1 (3.2) |  |
| Stevens–Johnson syndrome | 0 (0.0) | 0 (0.0) | 1 (3.2) | 1 (3.2) |  |
| Surgical and medical procedures | 1 (1.6) | 1 (1.6) | 0 (0.0) | 0 (0.0) |  |
| Hepatectomy | 1 (1.6) | 1 (1.6) | 0 (0.0) | 0 (0.0) |  |
| Vascular disorders | 1 (1.6) | 1 (1.6) | 0 (0.0) | 0 (0.0) |  |
| Hypertensive crisis | 1 (1.6) | 1 (1.6) | 0 (0.0) | 0 (0.0) |  |
| Abbreviations: AE, adverse event; ALT, alanine aminotransferase; AST, aspartate aminotransferase; CTCAE, Common Terminology Criteria for Adverse Events.  NOTE. Percentages are calculated using total number of patients per treatment as the denominator. MedDRA v17.0 was used for reporting. Only AEs with CTCAE grades equal to 1 to 5 are included. On-treatment AEs include the 28-day post-treatment period. AEs are sorted by frequency in the nintedanib 200 mg group.  ^a^One patient had one AE “metastases to central nervous system” with missing CTCAE grade. | | | | |  |
