# Supplementary material for: A multicentre, open-label, phase-I/randomised phase-II study to evaluate safety, pharmacokinetics, and efficacy of nintedanib vs. sorafenib in European patients with advanced hepatocellular carcinoma
Source: Br J Cancer. 2018 Mar 22;118(9):1162–8. doi: 10.1038/s41416-018-0051-8 (PMC5943284; doi:10.1038/s41416-018-0051-8)
Supplement: Supplementary file 13 — Supplementary Table S9(DOCX 32 kb) [file 41416_2018_51_MOESM13_ESM.docx]

| **Supplementary Table S9.** **Frequency of grade ≥3 adverse events of special interest by category and preferred term during the phase I portion** | | | | | | | | | |
| --- | --- | --- | --- | --- | --- | --- | --- | --- | --- |
|  | **Group I, *n* (%)** | | | | **Group II, *n* (%)** | | | | |
| **Nintedanib dose** | **100 mg bid (n=6)** | **150 mg bid (n=3)** | **200 mg bid (n=4)** | **Total (n=13)** | **50 mg bid (n=3)** | **100 mg bid (n=4)** | **150 mg bid (n=4)** | **200 mg bid (n=8)** | **Total (n=19)** |
| Abdominal pain | 2 (33.3) | 0 | 0 | 3 (23.1) | 2 (66.7) | 1 (25.0) | 0 | 2 (25.0) | 5 (26.3) |
| Abdominal pain | 2 (33.3) | 0 | 1 (25.0) | 3 (23.1) | 2 (66.7) | 1 (25.0) | 0 | 1 (12.5) | 4 (21.1) |
| Abdominal pain  upper | 0 | 0 | 1 (25.0) | 1 (7.7) | 1 (33.3) | 0 | 0 | 1 (12.5) | 2 (10.5) |
| Bleeding | 0 | 1 (33.3) | 0 | 1 (7.7) | 1 (33.3) | 0 | 0 | 2 (25.0) | 3 (15.8) |
| Bleeding varicose  vein | 0 | 0 | 0 | 0 | 0 | 0 | 0 | 1 (12.5) | 1 (5.3) |
| Gastrointestinal  haemorrhage | 0 | 1 (33.3) | 0 | 1 (7.7) | 0 | 0 | 0 | 0 | 0 |
| Hematemesis | 0 | 0 | 0 | 0 | 1 (33.3) | 0 | 0 | 1 (12.5) | 2 (10.5) |
| Melena | 0 | 0 | 0 | 0 | 1 (33.3) | 0 | 0 | 0 | 1 (5.3) |
| Cardiac arrest | 0 | 0 | 0 | 0 | 0 | 0 | 1 (25.0) | 0 | 1 (5.3) |
| Cardiac arrest | 0 | 0 | 0 | 0 | 0 | 0 | 1 (25.0) | 0 | 1 (5.3) |
| Cardiac arrhythmias | 0 | 1 (33.3) | 0 | 1 (7.7) | 0 | 0 | 1 (25.0) | 0 | 1 (5.3) |
| Syncope | 0 | 1 (33.3) | 0 | 1 (7.7) | 0 | 0 | 0 | 0 | 0 |
| Cardiac arrest | 0 | 0 | 0 | 0 | 0 | 0 | 1 (25.0) | 0 | 1 (5.3) |
| Dehydration | 0 | 0 | 0 | 0 | 1 (33.3) | 0 | 0 | 0 | 1 (5.3) |
| Dehydration | 0 | 0 | 0 | 0 | 1 (33.3) | 0 | 0 | 0 | 1 (5.3) |
| Diarrhoea | 0 | 0 | 1 (25.0) | 1 (7.7) | 0 | 0 | 0 | 0 | 0 |
| Diarrhoea | 0 | 0 | 1 (25.0) | 1 (7.7) | 0 | 0 | 0 | 0 | 0 |
| Fatigue | 0 | 0 | 0 | 0 | 0 | 0 | 1 (25.0) | 1 (12.5) | 2 (10.5) |
| Fatigue | 0 | 0 | 0 | 0 | 0 | 0 | 1 (25.0) | 1 (12.5) | 2 (10.5) |
| Hepatic failure | 0 | 1 (33.3) | 0 | 1 (7.7) | 2 (66.7) | 0 | 0 | 1 (12.5) | 4 (21.1) |
| Ascites | 0 | 1 (33.3) | 0 | 1 (7.7) | 2 (66.7) | 0 | 0 | 1 (12.5) | 3 (15.8) |
| Varices oesophageal | 0 | 0 | 0 | 0 | 1 (33.3) | 0 | 0 | 1 (12.5) | 2 (10.5) |
| Hypertension | 0 | 0 | 0 | 0 | 0 | 1 (25.0) | 0 | 0 | 1 (5.3) |
| Hypertension | 0 | 0 | 0 | 0 | 0 | 1 (25.0) | 0 | 0 | 1 (5.3) |
| Infection | 0 | 0 | 1 (25.0) | 1 (7.7) | 0 | 0 | 1 (25.0) | 1 (12.5) | 2 (10.5) |
| Biliary sepsis | 0 | 0 | 1 (25.0) | 1 (7.7) | 0 | 0 | 0 | 0 | 0 |
| Hepatitis B | 0 | 0 | 0 | 0 | 0 | 0 | 1 (25.0) | 0 | 1 (5.3) |
| Sepsis | 0 | 0 | 0 | 0 | 0 | 0 | 0 | 1 (12.5) | 1 (5.3) |
| Liver-related investigation | 2 (33.3) | 3 (100) | 0 | 5 (38.5) | 2 (66.7) | 1 (25.0) | 0 | 3 (37.5) | 6 (31.6) |
| Ascites | 0 | 1 (33.3) | 0 | 1 (7.7) | 2 (66.7) | 0 | 0 | 1 (12.5) | 3 (15.8) |
| AST increased | 0 | 1 (33.3) | 0 | 1 (7.7) | 0 | 0 | 0 | 2 (25.0) | 2 (10.5) |
| Blood ALP  increased | 0 | 1 (33.3) | 0 | 1 (7.7) | 0 | 1 (25.0) | 0 | 0 | 1 (5.3) |
| Blood bilirubin  increased | 2 (33.3) | 0 | 0 | 2 (15.4) | 0 | 0 | 0 | 1 (12.5) | 1 (5.3) |
| Nausea | 0 | 0 | 1 (25.0) | 1 (7.7) | 0 | 1 (25.0) | 0 | 0 | 1 (5.3) |
| Nausea | 0 | 0 | 1 (25.0) | 1 (7.7) | 0 | 1 (25.0) | 0 | 0 | 1 (5.3) |
| Peripheral neuropathies | 0 | 0 | 1 (25.0) | 1 (7.7) | 0 | 0 | 0 | 0 | 0 |
| Pain | 0 | 0 | 1 (25.0) | 1 (7.7) | 0 | 0 | 0 | 0 | 0 |
| Renal failure | 0 | 0 | 1 (25.0) | 1 (7.7) | 0 | 0 | 0 | 0 | 0 |
| Renal failure | 0 | 0 | 1 (25.0) | 1 (7.7) | 0 | 0 | 0 | 0 | 0 |
| Renal failure acute | 0 | 0 | 1 (25.0) | 1 (7.7) | 0 | 0 | 0 | 0 | 0 |
| Vomiting | 0 | 0 | 0 | 0 | 1 (33.3) | 1 (25.0) | 0 | 1 (12.5) | 3 (15.8) |
| Vomiting | 0 | 0 | 0 | 0 | 1 (33.3) | 1 (25.0) | 0 | 1 (12.5) | 3 (15.8) |
| Abbreviations: ALP, alkaline phosphatase; AST, aspartate aminotransferase.  Percentages are calculated using total number of patients per treatment as the denominator. MedDRA version 17.0 was used for reporting. Only adverse events with Common Terminology Criteria for Adverse Events grades 1–5 are included. On-treatment adverse events include the 28 day post-treatment period. No adverse events were observed for the following special interest categories: Anaphylactic reaction, Cardiac failure (tailored), Cutaneous adverse reactions, Febrile neutropenia, Gastrointestinal perforation, Hepatitis, Hypothyroidism, Interstitial lung disease, Myocardial infarction, Neutropenia, Non-gastrointestinal perforation, Osteonecrosis, Ovarian failure, Pulmonary hypertension, Renal failure, Sudden death, Venous thromboembolism. Some events contribute to more than one special interest category. Patients with such adverse events were counted in each of the Categories of adverse events of special interest (AESI) but were counted only once in the overall number of patients with AESI. | | | | | | | | | |
